# Computational Neural Modeling of Auditory Cortical Receptive Fields

**DOI:** 10.3389/fncom.2019.00028

**Published:** 2019-05-24

**Authors:** Jordan D. Chambers, Diego Elgueda, Jonathan B. Fritz, Shihab A. Shamma, Anthony N. Burkitt, David B. Grayden

**Affiliations:** ^1^NeuroEngineering Laboratory, Department of Biomedical Engineering, University of Melbourne, Parkville, VIC, Australia; ^2^Departamento de Patología Animal, Facultad de Ciencias Veterinarias y Pecuarias, Universidad de Chile, Santiago, Chile; ^3^Institute for Systems Research, University of Maryland, College Park, MD, United States; ^4^Laboratoire des Systèmes Perceptifs, École Normale Supérieure, Paris, France

**Keywords:** mathematical modeling, neural networks, auditory cortex, spectrotemporal receptive fields (STRFs), genetic algorithm

## Abstract

Previous studies have shown that the auditory cortex can enhance the perception of behaviorally important sounds in the presence of background noise, but the mechanisms by which it does this are not yet elucidated. Rapid plasticity of spectrotemporal receptive fields (STRFs) in the primary (A1) cortical neurons is observed during behavioral tasks that require discrimination of particular sounds. This rapid task-related change is believed to be one of the processing strategies utilized by the auditory cortex to selectively attend to one stream of sound in the presence of mixed sounds. However, the mechanism by which the brain evokes this rapid plasticity in the auditory cortex remains unclear. This paper uses a neural network model to investigate how synaptic transmission within the cortical neuron network can change the receptive fields of individual neurons. A sound signal was used as input to a model of the cochlea and auditory periphery, which activated or inhibited integrate-and-fire neuron models to represent networks in the primary auditory cortex. Each neuron in the network was tuned to a different frequency. All neurons were interconnected with excitatory or inhibitory synapses of varying strengths. Action potentials in one of the model neurons were used to calculate the receptive field using reverse correlation. The results were directly compared to previously recorded electrophysiological data from ferrets performing behavioral tasks that require discrimination of particular sounds. The neural network model could reproduce complex STRFs observed experimentally through optimizing the synaptic weights in the model. The model predicts that altering synaptic drive *between* cortical neurons and/or *bottom-up* synaptic drive from the cochlear model to the cortical neurons can account for rapid task-related changes observed experimentally in A1 neurons. By identifying changes in the synaptic drive during behavioral tasks, the model provides insights into the neural mechanisms utilized by the auditory cortex to enhance the perception of behaviorally salient sounds.

## Introduction

The auditory cortex utilizes a variety of processing strategies to enhance the perception of behaviorally-meaningful sounds in the presence of background noise. Rapid plasticity of receptive fields in primary (A1) cortical neurons is observed during behavioral tasks that require discrimination of particular sounds (Fritz et al., 2003, 2005a,b; Elhilali et al., 2004, 2007). This rapid, task-related change may enhance the ability to selectively attend to one acoustic feature or to one stream of sound in the presence of mixed sounds.

An essential property of acoustic signals is their temporal dynamics. Electrophysiological studies have shown that A1 neurons can encode the temporal structure of acoustic stimuli (Elhilali et al., 2004). A traditional view of auditory processing describes how a temporal sequence of sounds is distributed in the frequency domain along the auditory pathway from the basilar membrane to the cortex. Therefore, it is important to consider a sound's spectral and temporal features together.

The spectrotemporal receptive field (STRF) is a description an auditory neuron's input-to-output transformation encompassing both the spectral and temporal features. The STRFs of A1 neurons exhibit complex patterns that can undergo rapid, task-related changes (Fritz et al., 2003, 2005b; Elhilali et al., 2007). Complex patterns are observed, such as an increase in firing rate in response to increases in power at a certain frequency while decreasing firing rate in response to increases in power at an adjacent frequency (Fritz et al., 2005b). Rapid changes in the STRF are observed in A1 during task performance of ferrets trained to attend to a tone of any frequency (Fritz et al., 2003). Attending to a target tone consistently induced facilitative changes in the STRF at the location of the target tone for a conditioned avoidance Go-NoGo task, while in contrast, rapidly induce suppressive STRF changes at the target tone frequency in positive reinforcement Go-NoGo (David et al., 2012). However, the neural mechanisms by which cortical neurons dynamically change their STRFs in a matter of seconds remains unknown.

There have been several previous attempts to model the auditory cortex to investigate the role it plays in the perception of important sounds. For example, it has been shown that changes in STRFs can enhance discrimination between different sounds using mathematical filters (Mesgarani et al., 2010). However, using filters or similar macro mathematical processes (for example, Wrigley and Brown, 2004; Loebel et al., 2007; Grossberg and Kazerounian, 2011), neglects the fine temporal information that is encoded in the precise timing of action potential firing of neurons in the cortex (Elhilali et al., 2004). Other models that do contain fine temporal information (for example, Bendor, 2015) have not addressed the challenge of global auditory perception. Extending previous models to include fine temporal information has the potential to overcome the limitations of previous studies and allows for an investigation of the role of timing in the adaptive neural mechanisms utilized by the brain.

This study develops a neural network model that can produce realistic STRFs in response to a sound signal. The model consists of a cochlea model and a neural network model representing the A1 cortex. Action potentials in the neural network model were used to calculate the STRF. Mechanisms by which cortical neurons can change their STRF were investigated and directly compared to electrophysiological recordings. This model can reproduce complex STRFs observed experimentally. The model shows that synaptic drive between cortical neurons and/or synaptic drive from the cochlear model to the cortical neurons can account for rapid task-related changes exhibited by A1 neurons.

## Methods

**Figure 1:**
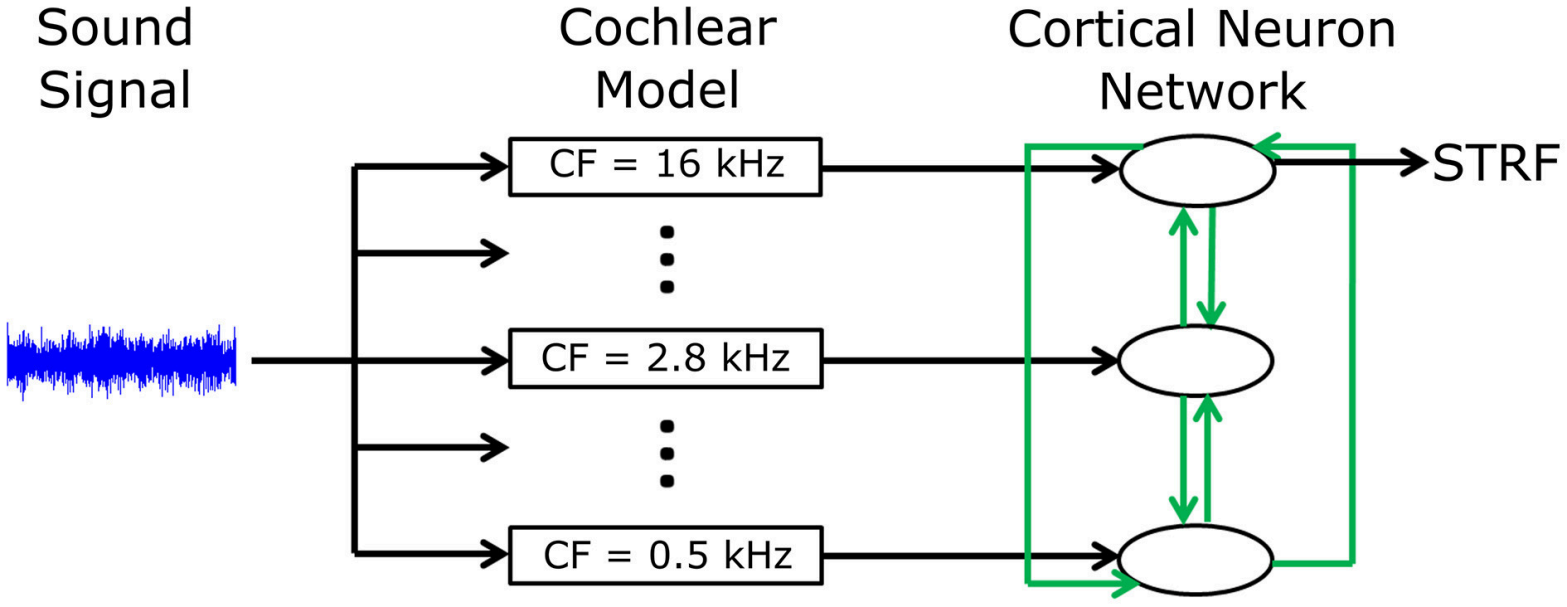
Schematic diagram of the general model structure. A sound signal was played into a model of the cochlea. There were 15 models in the cochlea, each with a different center frequency (CF) evenly spread along the logarithmic tonotopic axis. Integrate-and-fire neuron models were used to represent the neural networks in the A1 cortex. The output of the cochlear model excited or inhibited a single integrate-and-fire neuron model. All neurons were interconnected (indicated by the green arrows) with excitatory or inhibitory synapses of varying strengths. The action potentials from one cortical neuron model were used to calculate the STRF.

A phenomenological neural network model was developed to investigate mechanisms by which cortical neurons can change their spatiotemporal receptive fields. Figure 1 shows an overview of the model structure. A sound signal was sent into a model of the cochlea. The output of the cochlear model excited or inhibited integrate-and-fire neuron models to represent networks in the primary auditory cortex (A1). Each neuron in the network was tuned to a different frequency. All neurons were interconnected with excitatory or inhibitory synapses of varying strengths. Action potentials in one of the cortical neuron models was used to calculate the STRF using reverse correlation, which could be directly compared to electrophysiological recordings of real world, experimentally derived STRFs in ferrets (Fritz et al., 2003). The cortical neuron, from which the STRF was calculated, was chosen according to best frequency (defined as the frequency which produced largest spiking response at a given sound intensity) in the experimentally recorded STRF. A genetic algorithm was used to optimize the synaptic drive between neurons to produce STRFs and the behavioral changes in STRFs that matched experimentally recorded data.

### The Model

#### Sound Signals

Computer-generated sound signals were used to activate the mathematical model of the cochlea. The sound signals consisted of temporally orthogonal ripple combinations (Klein et al., 2000), called TORCs. The TORCs were synthesized from the general expression (Klein et al., 2000):

(1) $$\begin{array}{r} S\left(t,x\right)=\sum_{i=1}^{N}2a_{k_{i},l_{i}}\cos\left\{2\pi \left(\omega_{k_{i}}t+\varepsilon_{l_{i}}x\right)+\varphi_{k_{i}l_{i}}\right\}, \end{array}$$

where *N* is the number of distinct moving ripples, *t* is the time course of the stimuli, *x* is the bandwidth of the stimuli, *a*_*k, l*_ describes the amplitude, ω_*k*_ describes ripple velocity, ε_*l*_ describes the ripple frequency, and φ_*k, l*_ describes the phase of the stimulus components. The particular ripples chosen are parameterized by the list of indices *k* = [*k*_1_, *k*_2_, …, *k*_*N*_] ∈ (−∞, ∞) and *l* = [*l*_1_, *l*_2_, …, *l*_*N*_] ∈ (0, ∞).

To generate a STRF, a set of 30 TORCs were used. The set of TORCs had identical properties to TORCs used as acoustic stimuli in electrophysiological recordings. The TORCs had durations ranging over 1–2 s. Each TORC had a spectral profile that was the superposition of the envelopes of six ripples. Each ripple had a sinusoidal spectral profile with peaks spaced between 0 and 1.4 peaks per octave (ε_*l*_ in Equation 1) and the envelope drifted temporally along the logarithmic tonotopic frequency axis at a constant velocity ranging from 2–24 to 4–48 Hz (ω_*k*_ in Equation 1).

#### Cochlear Model

A model of the cochlea was used to convert the acoustic signal into neural activity. Fifteen auditory nerve fibers were simulated, with center frequencies evenly distributed along the logarithmic axis between 0.5 and 16 kHz. For each nerve, the auditory nerve model of Carney and colleagues was used (Tan and Carney, 2003; Zilany et al., 2009, 2014), so it will only be summarized here. There are two modes of basilar membrane excitation to the inner hair cell. The two modes are generated by two parallel filters. The first is a narrow-band chirp filter, which is responsible for low and moderate level responses. The second is linear, static, and broadly tuned, which is critical for producing transition regions and high-level effects. The responses from the two modes of the basilar membrane excitation are then added and passed through the inner hair cell low-pass filter followed by the inner hair cell-auditory nerve synapse model and discharge generator. The model responses are consistent with a wide range of physiological data from both normal and impaired ears for stimuli presented at levels spanning the dynamic range of hearing.

Version 5.2 of the auditory periphery model was used (Zilany et al., 2009) with modifications and updated simulation options (Zilany et al., 2014). Cat was used as the species option to produce good responses in the 12–16 kHz range, whereas the human option would show a decline in output above 12 kHz. A medium level of spontaneous activity and a variable noise type were chosen as these produced more robust responses when stimulating with TORCs. A normal setting for both inner and outer hair cell function was used.

#### Cortical Neuron Model

A standard integrate-and-fire neuron was used of the form,

(2) $$\begin{array}{r} \tau_{m}\frac{dv\left(t\right)}{dt}=\left(v_{rest}-v\left(t\right)\right)+I_{syn}\left(t\right)*R_{m}*\left(v\left(t\right)-v_{E_{I}}\right), \end{array}$$

where *v* is the membrane potential, *v*_*rest*_ is the resting membrane potential or equilibrium potential of the membrane leak, τ_*m*_ is the membrane time constant of the neuron, *R*_*m*_ is the membrane resistance, *I*_*syn*_ is the current resulting from the synaptic input into the neuron, and *v*_*E*_*I*__ is the driving force (or equilibrium potential) of the synaptic current.

Synaptic input from the Carney model and synaptic input from other cortical neurons (Figure 1) was modeled by an injected synaptic current with an alpha time course,

(3) $$\begin{array}{r} I_{syn}\left(t\right)=h\left(t\right)*g_{m}\frac{t}{\tau_{s}}e^{\frac{-t}{\tau_{s}}}, \end{array}$$

$$\text{where }h\left(t\right)=\{\begin{array}{c} 0\;ift\;\leq 0\\ 1\;if0<t \end{array}$$

where *t* is the time since the synaptic event, *g*_*m*_ is the maximum conductance, and τ_*s*_ is the time constant of the synapse.

The parameters values for Equations (2, 3) are given in Table 1.

**Table 1:** Numerical values used in the cortical neuron model.

**Parameter**	**Value**
τ_*m*_	10 ms
*v*_*rest*_	−70 mV
*R*_*m*_	4 MΩ
*v*_*E*_*I*__	−30 mV (excitatory)
	−90 mV (inhibitory)
*v*_*E*_*K*__	−90 mV
*g*_*m*_	2 μΩ
τ_*s*_	2 ms
Sampling rate	0.25 ms

*This table shows the numerical values used in the integrate-and-fire neuron model. These parameters appear in Equations (2, 3)*.

## Reverse Autocorrelation of the STRF

The STRF is a description of the auditory system's input-to-output transformation. It has the general form,

(4) $$\begin{array}{r} r\left(t\right)=\iint F_{STRF}\left(\tau ,x\right)*S\left(t-\tau ,x\right)d\tau dx, \end{array}$$

where *r* is a neuron response as a firing rate, *F*_*STRF*_ is the STRF functional, and *S* is the stimulus's dynamic spectrum.

The spectrotemporal reverse-correlation function *C*, is obtained by cross-correlating the dynamic spectrum of the stimulus with the measured response,

(5) $$\begin{array}{r} C\left(\tau ,x\right)=\frac{1}{T}\int_{0}^{T}S\left(t-\tau ,x\right)*r\left(t\right), \end{array}$$

where *x* is the frequency bands (and denotes the number of octaves above the lowest frequency). By inserting the STRF functional (Equation 4) directly into (Equation 5) and rearranging the terms, one obtains the spectrotemporal cross-correlation function,

(6) $$\begin{array}{r} C\left(\tau ,x\right)=\iint F_{STRF}{\left(\tau^{\prime },x^{\prime }\right)}^{*}\Phi \left(\tau -\tau^{\prime },x,x^{\prime }\right)d\tau^{\prime }dx^{\prime }+\varepsilon \left(\tau ,x\right), \end{array}$$

where ε is the portion of the measure response due to non-linear and random aspects of the system transformation not described by the STRF and Φ is given by

(7) $$\begin{array}{r} \Phi \left(\tau -\tau^{\prime },x^{\prime },x\right)≜\int S\;\left(t-\tau^{\prime },x^{\prime }\right)\;S\;(t-\tau ,x)dt. \end{array}$$

Here, Φ is a function that, in discrete channel interpretation, describes the cross-correlation between two channels *x* and x′ of the stimuli's dynamic spectrum. Thus, a single channel *x* of *C* is produced by the sum of the convolutions of every channel *x*′ of the STRF with the cross-correlation between the channels *x*′ and *x* of the stimulus. However, for both an ideal white noise dynamic spectrum and for a TORC, this expression reduces to a relatively simple two-dimensional convolution between the STRF and a spectrotemporal filter Φ(τ, *x*). For these two special cases, Φ depends only on the channel difference, x - x′, and is given by the autocorrelation of *S*.

### Optimization Algorithm

A genetic algorithm was used to optimize the synaptic drive between neurons to produce STRFs and the behavior-induced changes in STRF that matched experimentally recorded data. The neural network model contained 15 neurons, so the genetic algorithm optimized 255 parameters representing 225 synaptic connections between all 15 neurons, and 30 variables (2 per neuron) for the input from the cochlear model (15 for the strength of the input and 15 for the delay in transmission). All parameters were limited to a range of −5 to 5 and only integer values were used to reduce the probability of overfitting. A positive value for the synaptic drive resulted in an excitatory post-synaptic potential, whereas a negative value for the synaptic drive resulted in an inhibitory post-synaptic potential. The positive or negative values of the parameters for the delay in transmission were added to a baseline value to produce a transmission delay in the range of 0–50 ms.

The genetic algorithm followed a typical methodology. All parameters were assigned at random for the initial population of 1,000. The best 40 responses were classified as elite and passed directly to the next generation. The best 100 responses were used as parents for the next generation, where 480 were created from crossing-over parameters from parents and 480 were created from mutation of individual parents.

To determine the best responses, the STRF estimated from the action potentials in the cortical neuron network were compared with an experimentally recorded STRF by the cost function,

(8) $$\begin{array}{r} c=\sum_{i}^{N_{f}}\sum_{j}^{N_{t}}|\left(M_{STRF}\left(i,j\right)-E_{STRF}\left(i,j\right)\right)|*sig\left(E_{STRF}\left(i,j\right)\right), \end{array}$$

where *N*_*f*_ is the number of points along the frequency axis of the STRF, *N*_*t*_ is the number of points along the time axis of the STRF, *M*_*STRF*_ is the STRF calculated in the cortical neuron model, *E*_*STRF*_ is the experimentally recorded STRF, and *sig* is a function used to focus the genetic algorithm on the regions of the STRF that are significantly different from the remainder of the STRF. A point in the STRF was determined to be significantly different if it exceeded 3 standard deviations from the mean of the whole STRF,

(9) $$sig(x)=\{\begin{array}{cc} 1 & \text{if }x\geq 3\text{s}.\text{d}.\text{from mean}(x)\\ 0.1 & \text{if }x\;<3\text{s}.\text{d}.\text{from mean}(x) \end{array}$$

where *s*.*d*. is the standard deviation.

### Sensitivity Analysis

The genetic algorithm was used to optimize the 255 parameters in the mathematical model to reproduce the electrophysiologically recorded STRF in the model. This was performed for STRFs electrophysiologically recorded in the passive state and the behavioral states, where the ferret was actively listening for the target tone. A comparison between the model parameters optimized for the passive and behavioral states was performed by doing a sensitivity analysis on each parameter. The sensitivity analysis involved sequentially increasing and decreasing each parameter by one value from the optimal solution and determining the amount of change to the cost function (see Equation 8) and normalized over all 255 parameters. This sensitivity analysis quantified the effect of each parameter change upon the STRF for a given solution. The optimization by the genetic algorithm and sensitivity analysis was repeated five times for the same electrophysiologically recorded STRF.

To understand the changes occurring between the passive and behavioral states, a network diagram was generated containing the important parameters highlighted by the sensitivity analysis. A parameter was determined to be important if it was in the top two parameters of the sensitivity analysis for either the passive or behavioral states. Additional parameters were included to view the flow of information from the sound signal to the neuron from which the STRF was calculated to understand how the important parameters would be influencing the STRFs. The additional parameters included input from the cochlear model to neurons involved in the important parameters and synaptic connections between the neurons involved in the important parameters and the neuron from which the STRF was calculated from. Input from the cochlear model to the neuron from which the STRF was calculated was also included.

## Experimental Procedures

All experimental procedures were approved by the University of Maryland Institutional Animal Care and Use Committee (IACUC). Electrophysiological recordings from the A1 cortex of adult ferrets were performed with a behavioral paradigm requiring the ferrets to actively listen for a particular tone. This experimental procedure has been described in detail elsewhere (Fritz et al., 2003, 2005a,b), so will only be summarized here.

A stainless-steel head post was surgically implanted onto the skull and mounted with dental cement to stabilize the head for neurophysiological recordings. Craniotomies were made over auditory cortex, allowing microelectrodes to be inserted into A1. Location was based on stereotaxic coordinates and distinctive A1 neurophysiological characteristics such as latency, receptive field tuning, and position relative to the cortical tonotopic map.

Experiments were conducted in a sound attenuation chamber. Ferrets were trained on a tone detection task using a Go-NoGo conditional avoidance procedure (Klump, 1995; Fritz et al., 2003). In the passive state, the ferrets were awake and quiescent when the TORC stimuli were presented. In the behavioral state, ferrets licked water from a spout while listening to reference stimuli until they heard a target tone, whereupon the ferret learned to stop licking for a short period of time (400 ms) to avoid a mild shock. The same sets of TORCs and the same procedures for calculating the STRF from electrophysiologically recorded spikes were used in the animal experiments and the mathematical model described above.

## Results

A neural network model was developed to reproduce experimentally observed changes in STRFs of neurons in the A1 cortex. A sound signal was sent into a model of the cochlea, which provided input into integrate-and-fire neuron models to represent neural networks in the A1 cortex (Figure 1). The action potentials in one of the neuron models was used to calculate an STRF using reverse correlation, which could be directly compared to electrophysiological recording *in vivo*.

### Producing Anticipated STRFs

**Figure 2:**
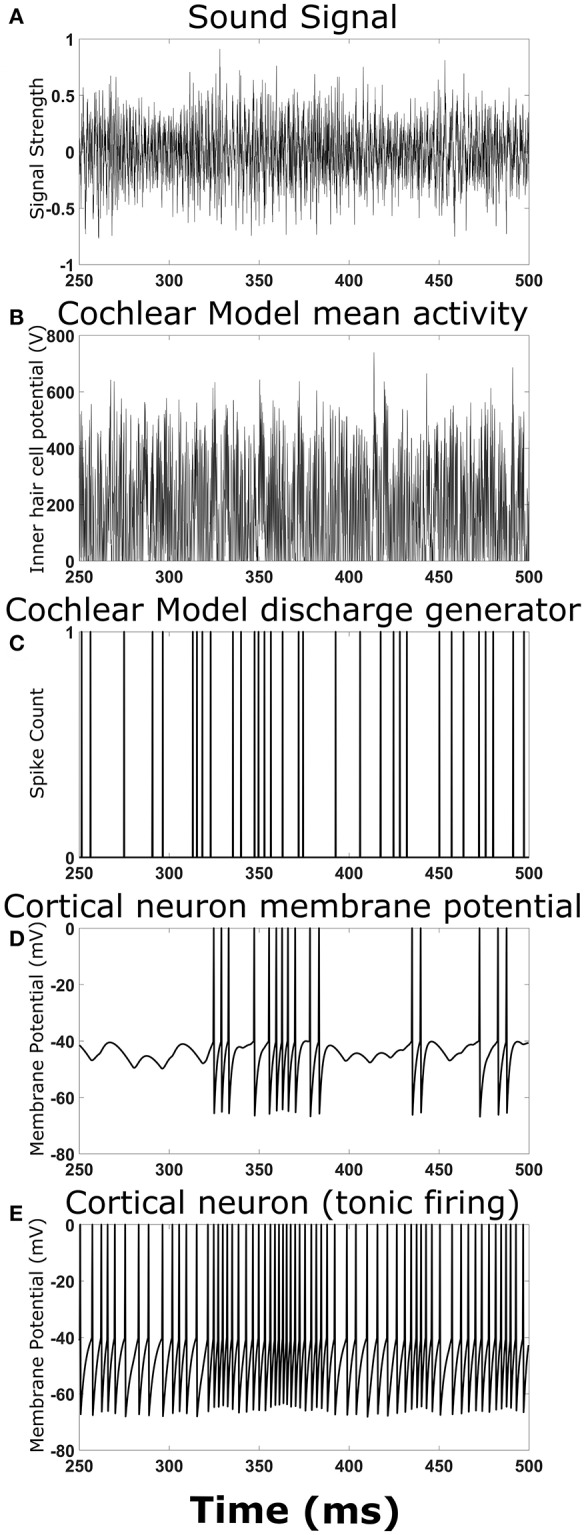
Output at different stages of the model. Examples of the signals and activity patterns for different components in the model. **(A)** An example of one cycle of a TORC sound signal sent into the model of the cochlea. **(B)** The mean activity rate in the model of the cochlea with a center frequency of 3 kHz. **(C)** Synaptic events that occur in the hair cell-auditory nerve synapse model and subsequent discharge generator in response to the mean activity rate in **(B)**. **(D)** Membrane potential trace from the cortical neuron model receiving the synaptic events from **(C)** with a 5 ms delay to represent transmission from the auditory nerve to the cortex. **(E)** Membrane potential trace from the cortical neuron model receiving the synaptic events from **(C)** with a delay in transmission. This panel differs from **(D)** because the cortical neuron model has a small constant drive to produce tonic firing.

To ensure the overall model structure was producing expected results, the cortical neural network model was reduced in complexity by removing synaptic connections between cortical neurons. Playing a simple chirp as the input sound signal produced activity in the cochlear model and cortical neuron model at the expected times (data not shown). Figure 2 shows an example TORC sound signal for one repetition (Figure 2A) and the resulting activity in the different stages of the model. The mean activity in the cochlear model (determined by the inner hair cell potential) fluctuated with the intensity of the sound signal (Figure 2B) and the resulting output from the discharge generator in the cochlear model was dependent on the mean activity (Figure 2C). Events in the discharge generator resulted in synaptic potentials in the cortical neuron model that were offset with a time delay corresponding to the delay in transmission from the cochlear model to the cortical neuron model (Figure 2D). When the events in the discharge generator were close enough together in time, summation of the synaptic potentials in the cortical neuron model could result in action potentials (Figure 2D). When investigating complex neural networks, more robust responses were observed with cortical neurons that had tonic firing of action potentials. Figure 2E shows a cortical neuron with tonic firing that received the exact same inputs as the cortical neuron in Figure 2D. The cortical neuron with tonic firing showed an increase in the action potential firing rate at times corresponding to the times of high activity in the discharge generator, as expected (Figure 2E). Therefore, when a TORC is played into the model, action potentials are observed in the cortical neurons as expected.

**Figure 3:**
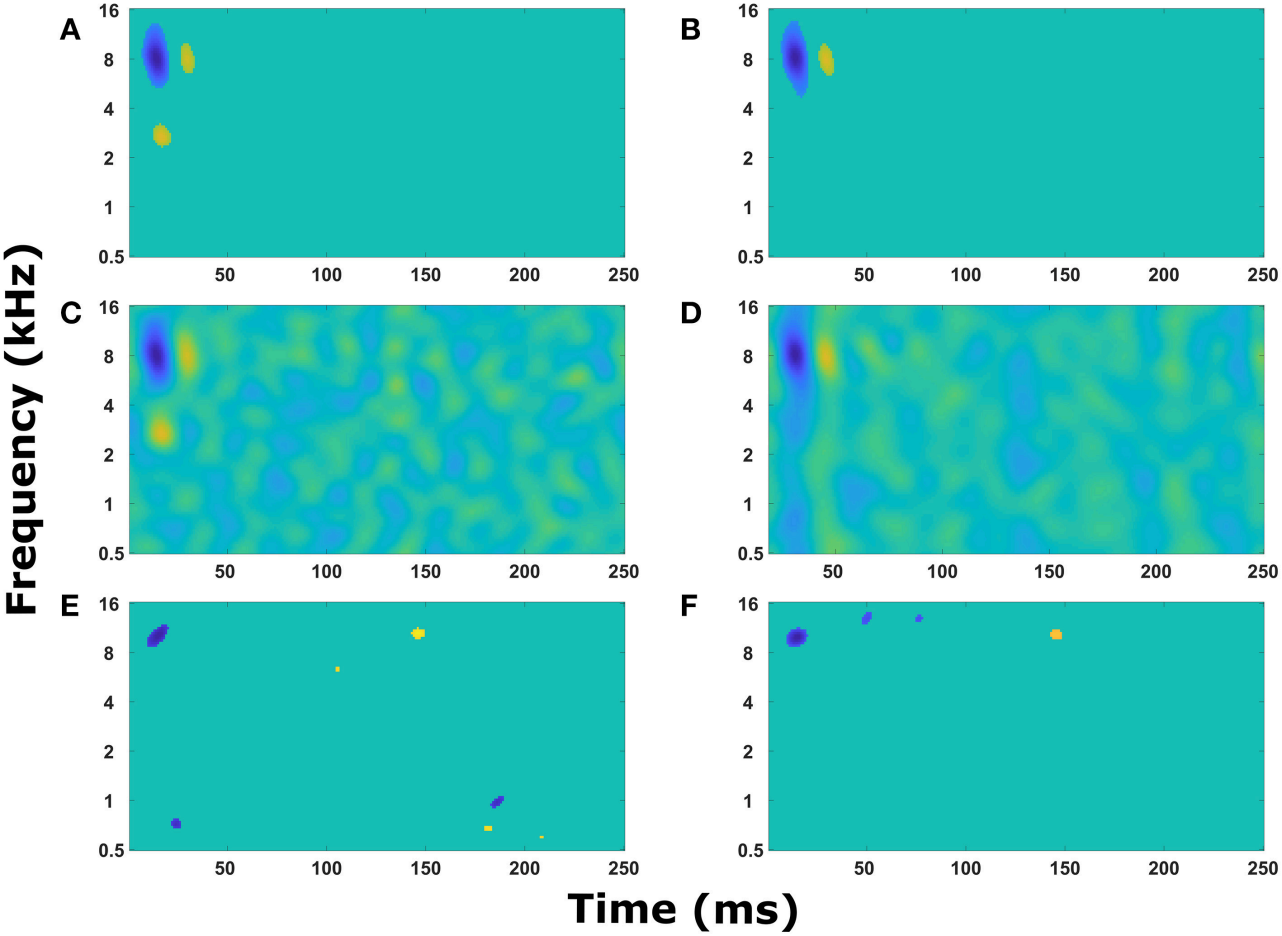
Simple STRF manipulations. Examples of the STRFs calculated from action potentials in the cortical neuron model for simple model structures to demonstrate the model is behaving as expected. **(A)** The cortical neuron received a simple excitatory connection from the cochlear model tuned with a center frequency of 5 kHz. **(B)** The cortical neuron received a simple excitatory connection from the cochlear model tuned with a center frequency of 2.5 kHz. **(C)** The cortical neuron received a simple inhibitory connection from the cochlear model tuned with a center frequency of 5 kHz. **(D)** The cortical neuron received a simple excitatory connection from the cochlear model tuned with a center frequency of 5 kHz, but the delay in transmission from the cochlea to the cortical neuron was increased from 5 to 55 ms. **(E)** The cortical neuron model contained two neurons, one that received input from the cochlear model tuned to 8 kHz and the second received input tuned to 3 kHz. The second neuron provided an inhibitory input into the first neuron. The STRF was calculated from the first neuron. **(F)** The cortical neuron model contained two neurons, one that received input from the cochlear model tuned to 8 kHz and the second received input tuned to 1.5 kHz. The second neuron provided an excitatory input into the first neuron. The STRF was calculated from the first neuron.

With a sound signal producing expected activity in the cochlear model and cortical neuron model, an STRF calculated from the action potentials in the cortical neuron model also produce expected results. When there were no synaptic connections between cortical neurons, each neuron produced a simple STRF with a simple excitatory region (Figure 3A). Altering the center frequency of tuning in the cochlear model (from 5 kHz in Figure 3A to 2.5 kHz in Figure 3B) moved the simple excitatory region to the expected frequency. Furthermore, switching the connection to inhibitory from the cochlear model to the cortical neuron model produced an inhibitory region (Figure 3C). Increasing the delay of transmission from the cochlear model to the cortical neuron model produced a time shift in the appearance of the excitatory region in the STRF (Figure 3D). These results demonstrate that the model is producing STRFs that accurately represent the model structure between the cochlear model and the cortical neuron model.

To ensure the cortical neuron network was also influencing STRF as expected, the cortical neuron network model was extended to include one synaptic connection from a cortical neuron receiving input from cochlear model tuned to a different frequency. This resulted in a second excitatory region (Figure 3F) or an inhibitory region (Figure 3E) depending on the type of synaptic connection. The transmission time from one cortical neuron to another caused a delay in the appearance of the second region in the STRF (Figures 3E,F). This delay is proportional to the difference in frequency of tuning in the cochlear model to represent the tonotopic organization observed in the A1 cortex.

In Figure 3, alternating regions of excitation and inhibition can be observed in the STRFs. Usually these regions are quite weak, but sometimes the regions can be strong (for example, the excitatory region at 3 kHz and 45 ms in Figure 3E). These alternating regions of excitation and inhibition are produced as a combination of the intrinsic properties of the neuron model (in particular resetting of the membrane potential after an action potential and the presence of an after-hyperpolarising potential, data not shown) and the properties of the TORCs. Reducing the noise within the model increases the appearance of these alternating regions, so they are obvious in Figure 3 where the model was simplified to ensure it was working properly.

### Reproducing Experimentally Observed STRFs

The excitatory and inhibitory regions in the STRFs calculated from this model of the auditory system can be manipulated by synaptic connections from other cortical neurons, synaptic drive from the cochlear model, and delay in transmission from the cochlear model to the cortical neuron model. Complex STRFs can be generated using different combinations of these manipulations. To test if this model can reproduce experimentally recorded STRFs, a genetic algorithm was used to optimize the STRF generated from the mathematical model with an experimentally recorded STRF.

**Figure 4:**
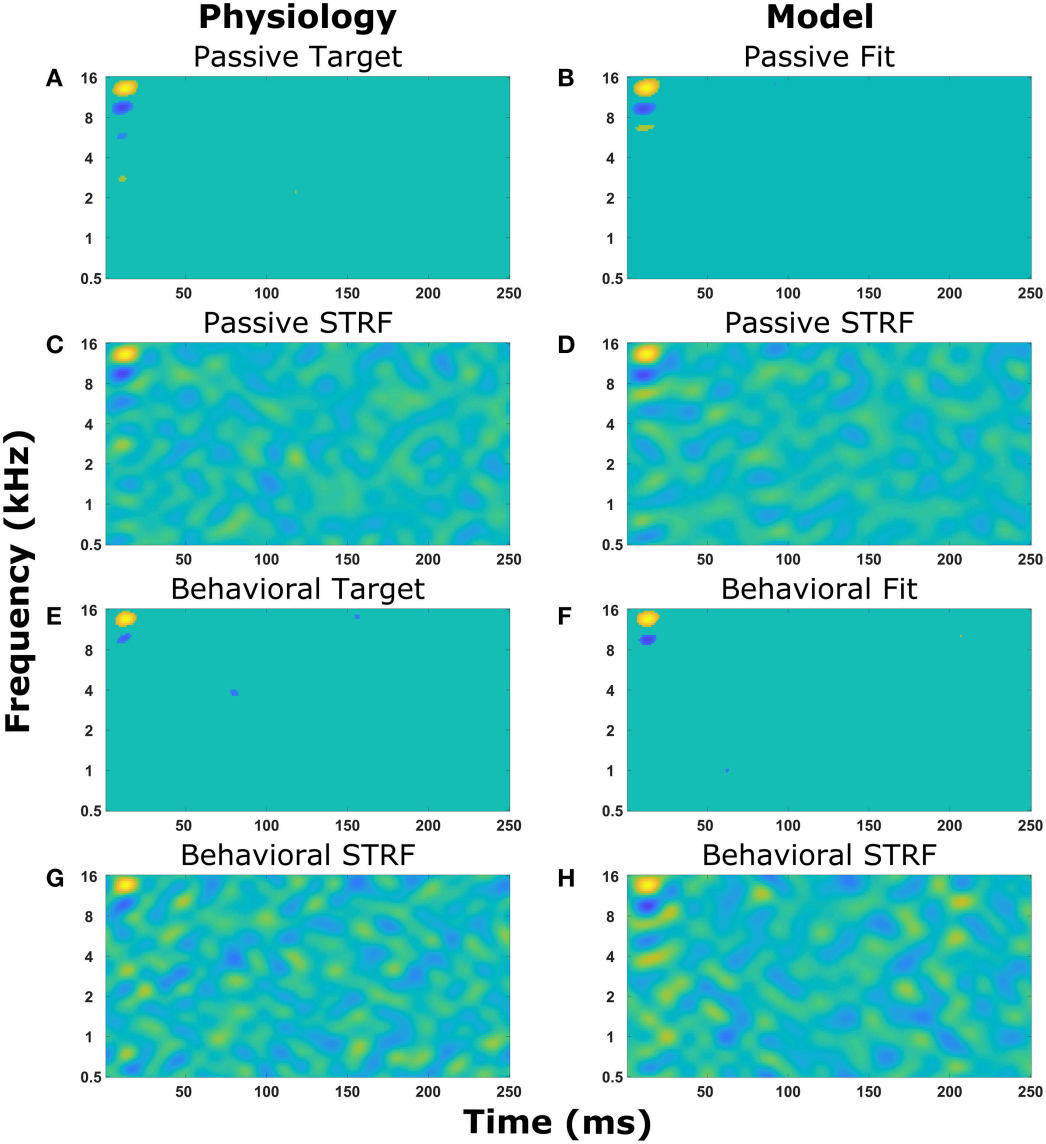
Reproducing experimentally observed STRFs. All the panels on the left-hand side are electrophysiological recordings from the A1 cortex of ferrets. All the panels on the right-hand side are output from the mathematical model. Identical sound signals were played to the ferrets and into the mathematical model. The mathematical model contained a network of neurons to represent the A1 cortex. All neurons were interconnected and the synaptic drive between them was optimized using a genetic algorithm to match the STRF recorded experimentally. **(A–D)** The passive state where the ferret was not actively listening for a tone. **(A,B)** Regions of the STRF that were significantly different (>3 standard deviations) from the mean value of the STRF. The passive target **(A)** from the physiological recordings was used in the cost function for the optimization. The passive fit **(B)** is the result of the genetic algorithm trying to reproduce **(A)**. **(C)** The STRF electrophysiologically recorded from a ferret. **(D)** The STRF calculated from the mathematical model. **(E–H)** The behavioral state where the ferret was actively detecting a tone. **(E,F)** Regions of the STRF that are significantly different (>3 standard deviations) from the mean value of the STRF. The behavioral target **(E)** was used in the cost function for the optimization. The behavioral fit **(F)** is the result of the genetic algorithm trying to reproduce **(E)**. **(G)** The STRF electrophysiologically recorded from a ferret. **(H)** The STRF calculated from the mathematical model.

Figure 4 displays the experimentally recorded STRFs and the STRFs generated from the computer model. We note that in physiological experiments, under both passive listening and active behavioral conditions, the same acoustic stimuli (i.e., broadband rippled noise combinations or TORCs, and target tones) were presented in identical order. To focus the genetic algorithm on important regions of the STRFs, points in the STRF more than three standard deviations from the mean value of all points in the STRF were emphasized (see Equations 8 and 9) and these physiologically recorded targets (Figures 4A,E) were closely matched by the model (Figures 4B,F). Furthermore, the STRFs electrophysiologically recorded and those generated from the mathematical model closely matched both the passive (Figures 4C,D) and the behavioral states (Figures 4G,H). This demonstrates that the optimization method and parameter space of the mathematical model enable the mathematical model to reproduce experimental observations. Repeating the optimization method with a different seed for the random number generator produce a different solution, which also closely matched the experimental observations. For each experimental observation, repeating the optimization five times produced five unique solutions (Figure S1), but often similar values of the cost function were produced over 100 generations of the genetic algorithm.

**Figure 5:**
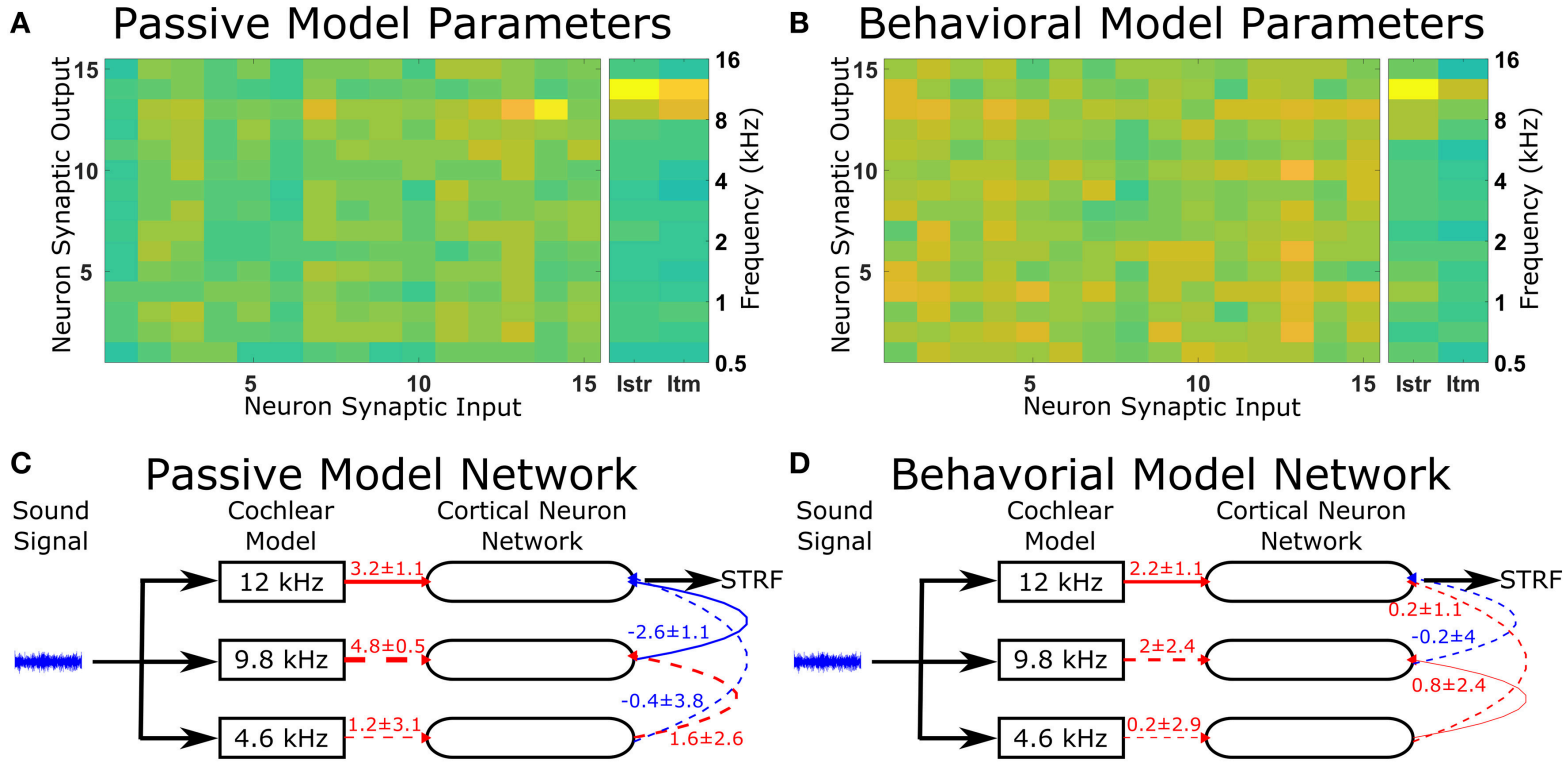
Changes in the network structure to reproduce experimental observations. The mathematical model can reproduce physiological recordings from the passive and behavioral states (Figure 4). To understand the changes in the network structure, the optimization was repeated five times and a sensitivity analysis was performed for each solution. **(A,B)** The average sensitivity analysis of the five solutions for both the passive **(A)** and behavioral **(B)** states. Both **(A,B)** contain two matrices, one 15-by-15 matrix and one 2-by-15 matrix. The 15-by-15 matrix represents the synaptic connections between all 15 neurons, with the y-axis representing the neuron number providing the synaptic output and the x-axis representing the neuron number receiving the synaptic. The 2-by-15 matrix represents the input from the cochlea model to the cortical neuron network, where Istr is the strength of the synaptic input and Itm is the length of the time delay for the synaptic input. The frequency axis on the far right hand side indicates the center frequency tuning for each neuron number. For clarity, if the sensitivity analyses were not in the top two values for either the passive or behavioral state, the value is not shown. **(C,D)** The network schematic diagram indicating the average values of the network parameters for the passive **(C)** and behavioral **(D)** states. Solid lines indicate that parameter had a high sensitivity (the two most sensitive parameters over five repetitions of the optimization) for that state, whereas dashed lines indicate the parameter was sensitive for the other state or were required to follow the network path from the sound signal to the neuron from which the STRF was calculated. Red lines indicate excitatory synaptic connections, blue lines indicate inhibitory synaptic connections, and the thicknesses of the lines indicate the strength of the synaptic connections. The numerical values presented for each line indicate the mean ± standard deviation for the five repetitions of the optimization.

The optimal solutions found by the genetic algorithm for the passive and behavioral states were compared to investigate the changes that are likely to occur from top-down attentional control. Since the genetic algorithm was optimizing 255 parameters, a sensitivity analysis was performed to highlight the parameters that were important in determining the behavior of the model in relation to the cost function. To account for the parameters highlighted by the sensitivity analysis due to chance in the complex multi-dimensional space, the genetic algorithm and sensitivity analysis were performed five times and the results averaged (Figure 5). The averaged sensitivity analysis indicated that three parameters were important in determining the network structure in the passive state (Figure 5A): (1) the input strength to the neuron from which the STRF was recorded, (2) the delay from the cochlea model to the neuron from which the STRF was recorded, and (3) the strength of the synaptic connection from the neuron tuned to the same frequency as the target tone to the neuron from which the STRF was recorded. Intuitively, these parameters correspond to the simplest network to generate the excitatory and inhibitory regions that are significantly different to the remaining parts of the STRF (Figure 4A). For the behavioral state, the average sensitivity analysis indicated that only one parameter was important in determining the network structure (Figure 5B), corresponding to the input strength to the neuron from which the STRF is recorded. Therefore, the sensitivity analysis allows a comparison of the average numerical values between the passive and behavioral states (Figures 5C,D). In this example, it can be seen that the behaviorally induced reduction in the STRF at ~8 kHz (Figures 4A,D) was reproduced in the mathematical model by reducing the strength of the inhibitory synaptic connection from neuron 13 (CF 10 kHz) to neuron 14 (CF 12 kHz) and by reducing the strength of input from the cochlea model to neuron 13 (CF 10 kHz). The network structures identified in Figure 5 do display a large amount of variation. Increasing the number of repetitions from 5 to 10 (see Figure S3) decreases the variation for some parameters, but increases the variation for other parameters. This indicates the variation is due to the genetic algorithm finding unique combinations of parameters to fit the physiological data.

### Reproducing More Experimentally Observed Changes in STRFs

**Figure 6:**
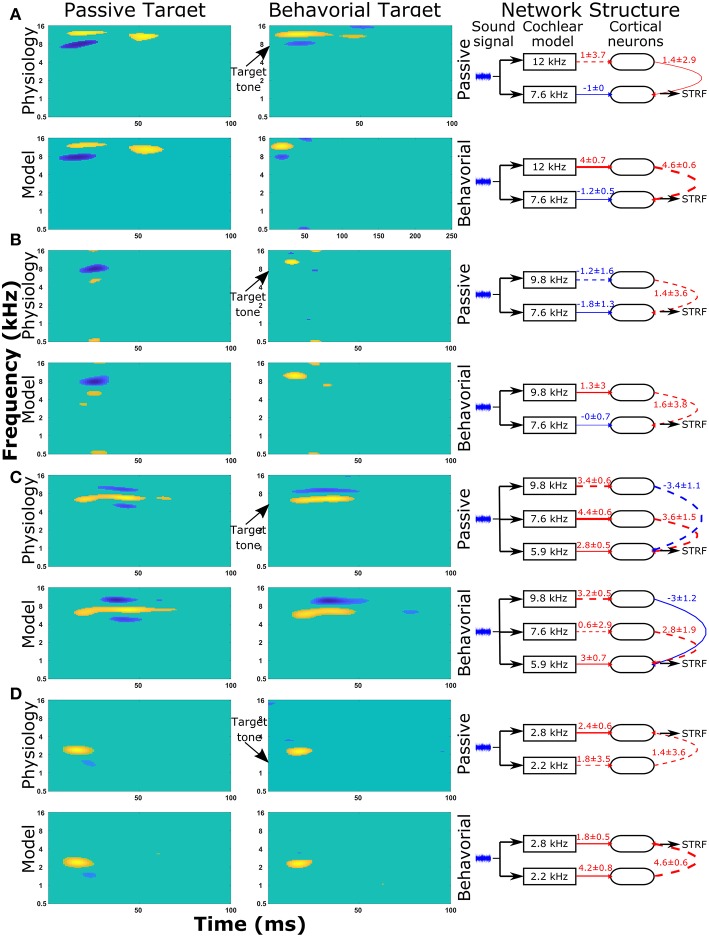
STRF and changes in the network structure for a further 4 single unit recordings. Physiological recordings were used to optimize the synaptic drive in a neural network model and sensitivity analyses of network structures were performed to highlight important parameters of four single unit recordings **(A–D)**. Within each panel, the first two columns display the regions of the STRF that were significantly different (>3 standard deviations) from the mean value of the STRF. The first column is for the passive state; the second column is the behavioral state. The first row of STRFs is electrophysiological recordings, while the second row is the outputs from the model. The third column displays the important network parameters for the passive and behavioral states. In the network schematic diagrams, solid lines indicate that parameters have a high sensitivity, whereas dashed lines indicate the parameter was not sensitive but is provided for comparison between the two network structures or to follow the pathway from sound signal to the neuron from which the STRF is calculated. Red lines indicate excitatory synaptic connections, blue lines indicate inhibitory synaptic connections, and the thicknesses of the lines indicate the strengths of the synaptic connections. The numerical values presented for each line indicate the mean ± standard deviation for the five repetitions of the optimization.

The same protocol (see section Reproducing Experimentally Observed STRFs) was used to predict the changes in network structure for a further nine single cell recordings from the A1 cortex of ferrets. The STRFs, significant components of the STRFs, sensitivity analyses, and important network parameters for the passive and behavioral states are provided for all 10 cells in the Supplementary Material (Figure S2). For each cell recording, the genetic algorithm was able to reproduce a close match to the experimentally recorded STRF for both the passive and behavioral states. Running the genetic algorithm five times for both the passive and behavioral states allowed sensitivity analyses to highlight important network parameters that were able to provide an explanation for the changes observed in the STRFs between passive and behavioral states (Figure 6 and Figure S2). Figure 6 provides a summary of four single unit recordings. For the first cell (Figure 6A), there was a reduction in the inhibitory region at ~8 kHz in the behavioral state compared to the passive state. This was reproduced in the network model by increasing the excitatory input from the neuron tuned to 12 kHz, which was producing the excitatory region in the STRF at this location (Figure 6A). For the second cell (Figure 6B), the inhibitory region at and below the target tone was converted to an excitatory region at the target tone during behavior. The network model reproduced this switch by changing the input to the neuron at the target tone from inhibitory input to excitatory input and removing the inhibitory input to the neuron tuned to a lower frequency (Figure 6B). The third example (Figure 6C) displayed a complex pattern of inhibitory regions above and below an excitatory region; during behavior the lower inhibitory region was abolished. The sensitivity analysis indicated the important network parameters were determining the excitatory region and the higher frequency inhibitory region, so these network parameters were maintained between the passive and behavioral states (Figure 6C). Intuitively, this makes sense because the excitatory and higher inhibitory regions are larger than the lower inhibitory region, so they would have a larger influence on the cost function. In the fourth example (Figure 6D), the inhibitory region at ~1.5 kHz was abolished in the behavioral state. The network model reproduced this removal of the inhibitory region by increasing the strength of the excitatory region (Figure 6D), which reduces the magnitude and significance of the inhibitory connection from the lower frequency.

## Discussion

Temporal dynamics are a key component of acoustic signals and neurons in the primary auditory cortex can detect the temporal structure of acoustic signals (Elhilali et al., 2004). The cochlea and auditory pathway distribute sounds in the frequency domain from the cochlear basilar membrane to the auditory cortex. Therefore, it is important to consider a sound's spectral and temporal features together. Spectrotemporal receptive fields (STRFs) combine both the spectral and temporal features of the auditory system. Previous studies have shown that the STRFs of A1 neurons display rapid plasticity during behavioral tasks requiring discrimination of particular sounds (Fritz et al., 2003, 2005b; Elhilali et al., 2007). However, the neural mechanisms underlying changes in a neuron's STRFs are yet to be elucidated. In this study, a neural network model was developed to investigate mechanisms by which cortical neurons can change their receptive fields. This model can reproduce complex STRFs observed experimentally and demonstrates that altering the synaptic drive between cortical neurons and/or synaptic drive from the cochlear model to cortical neurons can account for the rapid-task related changes displayed by A1 neurons.

The mathematical model presented here comprised a cochlear model and a cortical neuron network. A sound signal was sent into a model of the cochlea. The cochlear model consisted of 15 auditory nerve fiber models, previously developed and published by Carney and colleagues (Tan and Carney, 2003; Zilany et al., 2009, 2014). Each of the auditory nerve models had a different center frequency evenly distributed along a logarithmic-tonotopic axis. The synaptic output from the cochlear model excited or inhibited one integrate-and-fire neuron model. There were 15 integrate-and-fire neuron models to represent networks in the A1 cortex. All neurons were interconnected with excitatory or inhibitory synapses of varying strengths. Action potentials of one of the cortical neuron models was used to calculate the STRF using reverse correlation, which could be directly compared to electrophysiological recordings of the STRF a ferret (Fritz et al., 2003). A genetic algorithm was used to optimize the synaptic drive between neurons to produce STRFs and the behavioral changes in STRFs that matched experimentally recorded data.

The results demonstrate that this simple phenomenological model can produce complex STRFs similar to those observed experimentally. The genetic algorithm was able to optimize the synaptic drive in the cortical neural network to ensure a close match between an electrophysiologically recorded STRF and the STRF calculated in the mathematical model. A genetic algorithm was used because there were a large number of variables, the cost function relating the fitting the STRFs to experimental data was not a smooth function and as a method of avoid local optimal solutions in a neural network with a large number of possible synaptic pathways. This optimization worked for both the passive and behavioral states, thereby allowing a comparison between network parameters for both states. Since the genetic algorithm was optimizing 255 parameters and the network behavior could be highly influenced by combinations of multiple parameters, the genetic algorithm optimization and sensitivity analyses were repeated five times and averaged. For each repetition, the optimization algorithm produced a unique set of parameters that produced an STRF similar to those observed experimentally. While the network parameters displayed variations between repetitions, the sensitivity analysis highlighted network parameters whose influence over the network behavior was preserved multiple times. The sensitivity analysis was also important for comparisons of parameter values between the passive and behavioral states where the numerical value showed little change. Traditional statistical methods would deem that no change has occurred, suggesting the parameter is not important in the switch from passive to behavioral states. However, the sensitivity analysis could show that a parameter was indeed important in determining the properties of the neural network for one or both states, even if there was no change in that parameter. Therefore, this process is able to predict the important network parameters and their changes, or no change, in the passive and behavioral states.

This work has demonstrated that changes observed in STRF of neurons in the A1 cortex for behavioral tasks can be accounted for by changes in the synaptic drive between cortical neurons and/or synaptic drive from the cochlear model to the cortical neurons. The changes in the STRF between passive and behavioral states can occur with seconds to minutes (Fritz et al., 2003; Lu et al., 2017) and can dissipate rapidly or remain stable for a long time (Fritz et al., 2003). There are several mechanisms that can potentially change the synaptic drive between two neurons with a very rapid time course (such as synaptic potentials, spike-timing dependent plasticity (STDP), changes in dendritic spine shape as well as activity dependent depression) or in a manner that remains stable for an extended period of time (such as synaptogenesis, or long-term changes mediated by intracellular second messenger systems). A major advantage of producing a spiking neuron model to reproduce experimentally observed changes in the STRF is that these mechanisms for rapid changes or longer stability can be readily investigated by evoking the appropriate mechanisms within a single neuron. These investigations will require further challenging studies, such as intracellular recordings from individual A1 neurons in a behaving animal.

In this study, the experimental paradigm produced a positive change in the STRF at the target tone, which has been previously reported (Fritz et al., 2003, 2005a,b; Elhilali et al., 2004, 2007). In individual examples reproduced in this mathematical model, the model predicted the increased excitation in the behavioral STRF at the target tone could arise from reduced inhibitory output from the neuron at the target tone, increased excitatory synaptic drive from a region different to the target tone (which can reduce the influence of inhibition from the target tone; e.g., cell 10, Figure S2), or by reduced synaptic drive from the cochlear model to the neuron at the target tone. These different mechanisms could potentially be distinguished by further experimental studies. For example, distinguishing between increased excitatory drive and decreased inhibitory drive could be accomplished by pharmacological intervention or by directly recording neuronal membrane potential. Similarly, a bottom-up change in synaptic drive from the cochlear model to the cortical neurons could be distinguished from cortical neuron to cortical neuron interactions through localized pharmacological agonists or blockers for relevant neurotransmitters or neuromodulators. Such experiments require technical advances for pharmacological, optogenetic manipulations, and intracellular or patch-clamp recordings from A1 neurons in animal engaged in behavioral tasks such as the ones used in this study. The increased synaptic drive from the cochlear model could be measured experimentally by examining whether there was enhanced thalamic input to A1 neurons. Increase in thalamic drive to auditory cortex could arise from a variety of mechanisms, including increased thalamic firing or disinhibition of inhibitory circuits in A1 (for example, Letzkus et al., 2011).

The current model predicts that rapid task-related changes in A1 STRFs occur at the synaptic level by changing the weights of task-relevant synaptic inputs to A1 neurons; however, the mechanisms for this plasticity are not yet known. Neuroanatomical studies have shown the existence of diffuse and widespread cholinergic projections (that modify synaptic behavior) from the Nucleus Basalis to A1 that are likely to play an important role in neuroplasticity (Goard and Dan, 2009; Leach et al., 2013; Pinto et al., 2013; Bajo et al., 2014; Zhang et al., 2014, 2016). This projection pattern would suggest that cholinergic modulation during attentional tasks should produce uniform changes across the entire A1 cortex. However, our experimental results demonstrate highly selective attentional effects, and in our model, the pattern of network changes between passive and behavioral states was variable and complex, involving increases, no change, and/or decreases in synaptic drive at different synapses. The solutions generated by the model predict that the effect of top-down control from higher executive brain regions via cholinergic activation from Nucleus Basalis can influence the synaptic drive in A1 cortex in a specific fashion, perhaps by selectively modulating A1 synapses and neurons “tagged” by recent “target stimulus” activation. A previous model of cholinergic modulation of A1 suggests differential effects on the receptive fields of cortical neurons, depending on cholinergic receptors and site of action (thalamocortical or intracortical) (Soto et al., 2006). Another possibility is that there is focal top-down control from higher brain regions that target the task-relevant subset of synaptic sites in A1 cortex. Our spiking neuron network model of the auditory receptive fields provides a platform to test these and other possible mechanisms of top-down control during behavioral tasks (Zhang et al., 2014, 2016).

In addition to using the Carney and colleagues model for the auditory nerve fibers (Tan and Carney, 2003; Zilany et al., 2009, 2014), we also tested the early auditory processing model of Shamma and colleagues (Chi et al., 2005) and gamma-tone filter banks (Johannesma, 1972). All three types of models produced qualitatively similar results. This indicates that the important process performed by the cochlea model in this work is band-pass filtering to break up the sound signal into the frequency components. Such band-pass filtering is present in the three cochlear models tested here. Other features present in some of these cochlear models and not others (for example, synaptic adaptation in the Carney model or lateral inhibition in the Shamma model) do not have a significant effect on the qualitative results observed in the model presented in this study. The model of Carney and colleagues had the advantage of better responses in the higher frequency ranges (12–16 kHz) used in this mathematical model. The better responses observed in these ranges may have been due to the high frequency range of the cat, whereas other cochlear models based on humans have responses that start to drop off at frequencies above 12 kHz, as did the model of Carney and colleagues when using the human parameters. The model of Carney and colleagues also had the advantage of providing a discrete synaptic drive into the integrate-and-fire neuron model compared to a continuous output provided by other cochlear models. A discrete synaptic drive is a more realistic response, but does create an additional source of noise in the system because the amplitudes are driving a Poisson process.

The model presented here is missing many steps from the cochlear model to the neural network model in the A1 cortex. As stated in the previous paragraph, qualitatively similar results can be observed using the cochlear model of Shamma and colleagues (Chi et al., 2005), which incorporates a limited amount of early processing such as lateral inhibition. However, obviously, given the multiple neuroanatomical stages for auditory information processing between cochlea and cortex (including cochlear nucleus, laminar lemniscus, inferior colliculus, thalamus), our model is oversimplified. Nevertheless, these results indicate that it is not critical to include early processing strategies to reproduce the electrophysiologically recorded STRFs. However, this model was tested against neurons that showed a change in their STRF during behavioral tasks and ~70% of neurons show a change in their STRF during behavioral tasks (Fritz et al., 2003). It is also possible that initializing an A1 cortical neuron network where all neurons are interconnected allows the optimization to include early auditory processing as well as A1 cortical processing. Our results indicate that it is not necessary to incorporate early auditory processing to reproduce the experimental observations in the model presented here.

Furthermore, our model included two variables for each neuron to describe the strength and timing of the connections from the cochlear to the auditory cortex. These variables were included in the optimization and the sensitivity analyses indicated these connections from the cochlear to the auditory cortex are significant at times. Therefore, bottom-up information from band-pass filtering can be important in this model of STRFs, but the inclusion of exclusion of early processing strategies does not have a significant effect in this model of STRFs.

In conclusion, this study has produced a mathematical model that can replicate complex STRFs observed in response to the same sound signals. The model demonstrates that synaptic drive between cortical neurons can account for rapid task-related changes exhibited by A1 neurons. These results lay a foundation for future extensions and elaborations of this model to include top-down control from higher brain regions, and a more detailed investigation into the multiple cellular mechanisms and neuronal receptive field plasticity utilized by the brain during sound discrimination tasks.

## Ethics Statement

All experimental procedures were approved by the University of Maryland Animal Care and Use Committee.

## Author Contributions

JC, JF, SS, AB, and DG conceived and planned the experiments. JC planned and carried out the simulations and analysis. DE performed all animal experiments. JC took the lead in writing the manuscript. All authors contributed to the interpretation of results and provided critical feedback and helped shape the research, analysis, and manuscript.

### Conflict of Interest Statement

The authors declare that the research was conducted in the absence of any commercial or financial relationships that could be construed as a potential conflict of interest.

## References

[B1] Bajo V. M. Leach N. D. Cordery P. M. Nodal F. R. King A. J. (2014). The cholinergic basal forebrain in the ferret and its inputs to the auditory cortex. Eur. J. Neurosci. 40, 2922–2940. 10.1111/ejn.12653 24945075 PMC4215603

[B2] Bendor D. (2015). The role of inhibition in a computational model of an auditory cortical neuron during the encoding of temporal information. PLoS Comput. Biol. 11:e1004197. 10.1371/journal.pcbi.1004197 25879843 PMC4400160

[B3] Chi T. Ru P. Shamma S. A. (2005). Multiresolution spectrotemporal analysis of complex sounds. J. Acoust. Soc. Am. 118, 887–906. 10.1121/1.1945807 16158645

[B4] David S. V. Fritz J. B. Shamma S. A. (2012). Task reward structure shapes rapid receptive field plasticity in auditory cortex. Proc. Natl. Acad. Sci. U.S.A. 109, 2144–2149. 10.1073/pnas.1117717109 22308415 PMC3277538

[B5] Elhilali M. Fritz J. B. Chi T. S. Shamma S. A. (2007). Auditory cortical receptive fields: stable entities with plastic abilities. J. Neurosci. 27, 10372–10382. 10.1523/JNEUROSCI.1462-07.2007 17898209 PMC6673154

[B6] Elhilali M. Fritz J. B. Klein D. J. Simon J. Z. Shamma S. A. (2004). Dynamics of precise spike timing in primary auditory cortex. J. Neurosci. 24, 1159–1172. 10.1523/JNEUROSCI.3825-03.2004 14762134 PMC6793586

[B7] Fritz J. Elhilali M. Shamma S. (2005a). Active listening: task-dependent plasticity of spectrotemporal receptive fields in primary auditory cortex. Hear Res. 206, 159–176. 10.1016/j.heares.2005.01.015 16081006

[B8] Fritz J. Shamma S. Elhilali M. Klein D. (2003). Rapid task-related plasticity of spectrotemporal receptive fields in primary auditory cortex. Nat. Neurosci. 6, 1216–1223. 10.1038/nn1141 14583754

[B9] Fritz J. B. Elhilali M. Shamma S. A. (2005b). Differential dynamic plasticity of A1 receptive fields during multiple spectral tasks. J. Neurosci. 25, 7623–7635. 10.1523/JNEUROSCI.1318-05.2005 16107649 PMC6725393

[B10] Goard M. Dan Y. (2009). Basal forebrain activation enhances cortical coding of natural scenes. Nat. Neurosci. 12, 1444–1449. 10.1038/nn.2402 19801988 PMC3576925

[B11] Grossberg S. Kazerounian S. (2011). Laminar cortical dynamics of conscious speech perception: neural model of phonemic restoration using subsequent context in noise. J. Acoust. Soc. Am. 130, 440–460. 10.1121/1.3589258 21786911

[B12] Johannesma P. I. (1972). “The Pre-response Stimulus Ensemble of neurons in the cochlear nucleus,” in: Symposium on Hearing Theory. Eindhoven: IPO Eindhoven.

[B13] Klein D. J. Depireux D. A. Simon J. Z. Shamma S. A. (2000). Robust spectrotemporal reverse correlation for the auditory system: optimizing stimulus design. J. Comput. Neurosci. 9, 85–111. 10.1023/A:1008990412183 10946994

[B14] Klump G. M. (1995). Methods in Comparative Psychoacoustics. Basel ; Boston, MA: Birkhäuser Verlag, c1995.,. 10.1007/978-3-0348-7463-2

[B15] Leach N. D. Nodal F. R. Cordery P. M. King A. J. Bajo V. M. (2013). Cortical cholinergic input is required for normal auditory perception and experience-dependent plasticity in adult ferrets. J. Neurosci. 33, 6659–6671. 10.1523/JNEUROSCI.5039-12.2013 23575862 PMC3682393

[B16] Letzkus J. J. Wolff S. B. Meyer E. M. Tovote P. Courtin J. Herry C.. (2011). A disinhibitory microcircuit for associative fear learning in the auditory cortex. Nature 480, 331–335. 10.1038/nature10674 22158104

[B17] Loebel A. Nelken I. Tsodyks M. (2007). Processing of sounds by population spikes in a model of primary auditory cortex. Front. Neurosci. 1, 197–209. 10.3389/neuro.01.1.1.015.2007 18982129 PMC2570089

[B18] Lu K. Xu Y. Yin P. Oxenham A. J. Fritz J. B. Shamma S. A. (2017). Temporal coherence structure rapidly shapes neuronal interactions. Nat. Commun. 8:13900. 10.1038/ncomms13900 28054545 PMC5228385

[B19] Mesgarani N. Fritz J. Shamma S. (2010). A computational model of rapid task-related plasticity of auditory cortical receptive fields. J. Comput. Neurosci. 28, 19–27. 10.1007/s10827-009-0181-3 19711179 PMC3973422

[B20] Pinto L. Goard M. J. Estandian D. Xu M. Kwan A. C. Lee S. H.. (2013). Fast modulation of visual perception by basal forebrain cholinergic neurons. Nat. Neurosci. 16, 1857–1863. 10.1038/nn.3552 24162654 PMC4201942

[B21] Soto G. Kopell N. Sen K. (2006). Network architecture, receptive fields, and neuromodulation: computational and functional implications of cholinergic modulation in primary auditory cortex. J. Neurophysiol. 96, 2972–2983. 10.1152/jn.00459.2006 16899641

[B22] Tan Q. Carney L. H. (2003). A phenomenological model for the responses of auditory-nerve fibers. II. Nonlinear tuning with a frequency glide. J. Acoust. Soc. Am. 114(4 Pt 1), 2007–2020. 10.1121/1.1608963 14587601

[B23] Wrigley S. N. Brown G. J. (2004). A computational model of auditory selective attention. IEEE Trans. Neural. Netw. 15, 1151–1163. 10.1109/TNN.2004.832710 15484891

[B24] Zhang S. Xu M. Chang W. C. Ma C. Hoang Do J. P. Jeong D.. (2016). Organization of long-range inputs and outputs of frontal cortex for top-down control. Nat. Neurosci. 19, 1733–1742. 10.1038/nn.4417 27749828 PMC5127741

[B25] Zhang S. Xu M. Kamigaki T. Hoang Do J. P. Chang W. C. Jenvay S.. (2014). Selective attention. Long-range and local circuits for top-down modulation of visual cortex processing. Science 345, 660–665. 10.1126/science.1254126 25104383 PMC5776147

[B26] Zilany M. S. Bruce I. C. Carney L. H. (2014). Updated parameters and expanded simulation options for a model of the auditory periphery. J. Acoust. Soc. Am. 135, 283–286. 10.1121/1.4837815 24437768 PMC3985897

[B27] Zilany M. S. Bruce I. C. Nelson P. C. Carney L. H. (2009). A phenomenological model of the synapse between the inner hair cell and auditory nerve: long-term adaptation with power-law dynamics. J. Acoust. Soc. Am. 126, 2390–2412. 10.1121/1.3238250 19894822 PMC2787068

