# Supplementary material for: Computational Neural Modeling of Auditory Cortical Receptive Fields
Source: Front Comput Neurosci. 2019 May 24;13:28. doi: 10.3389/fncom.2019.00028 (PMC6543553; doi:10.3389/fncom.2019.00028)
Supplement: Supplementary file 1 [file Data_Sheet_1.PDF]

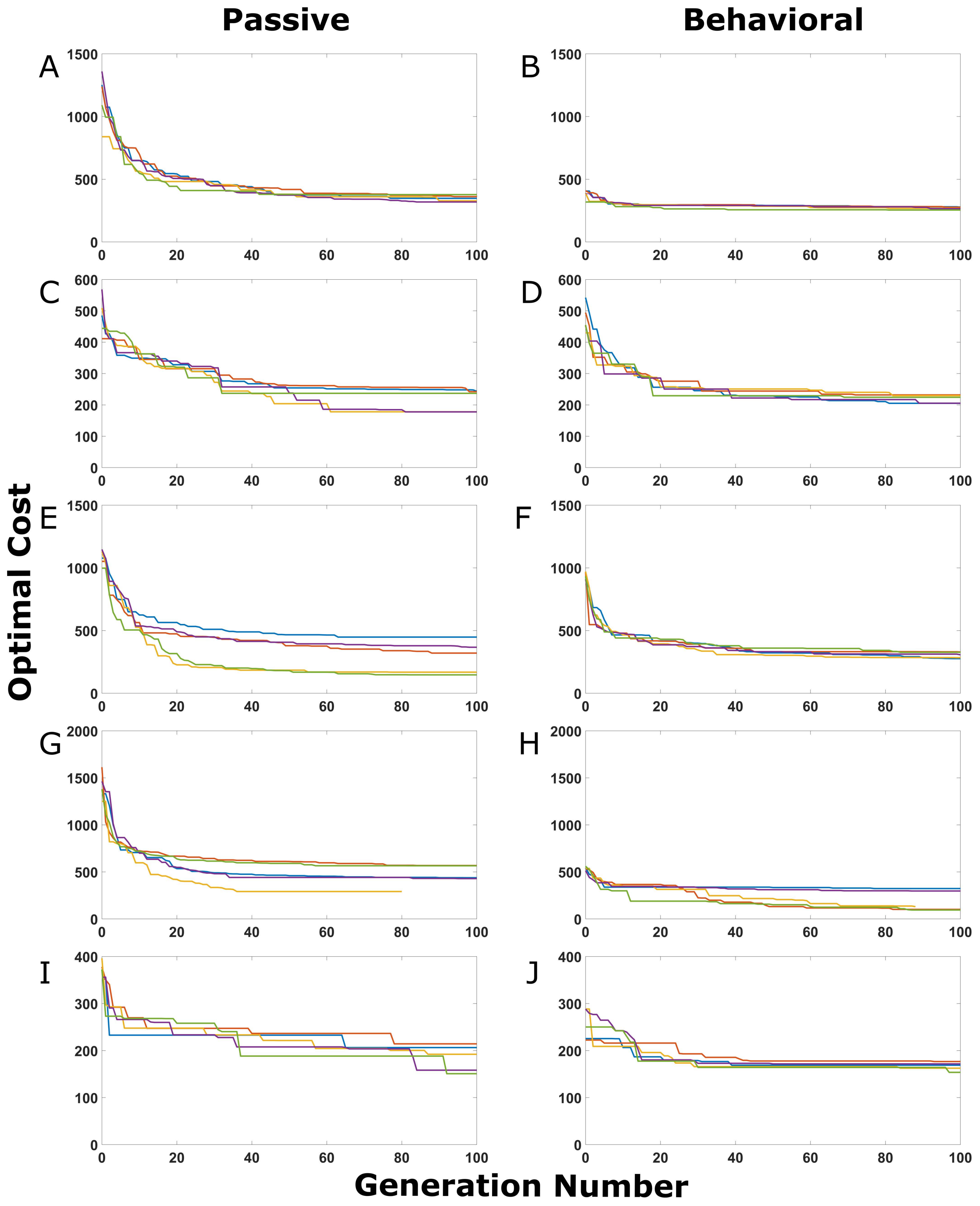
Passive
Behavioral
A
B
C
D
E
F
G
H
I
J
Optimal Cost
Generation Number

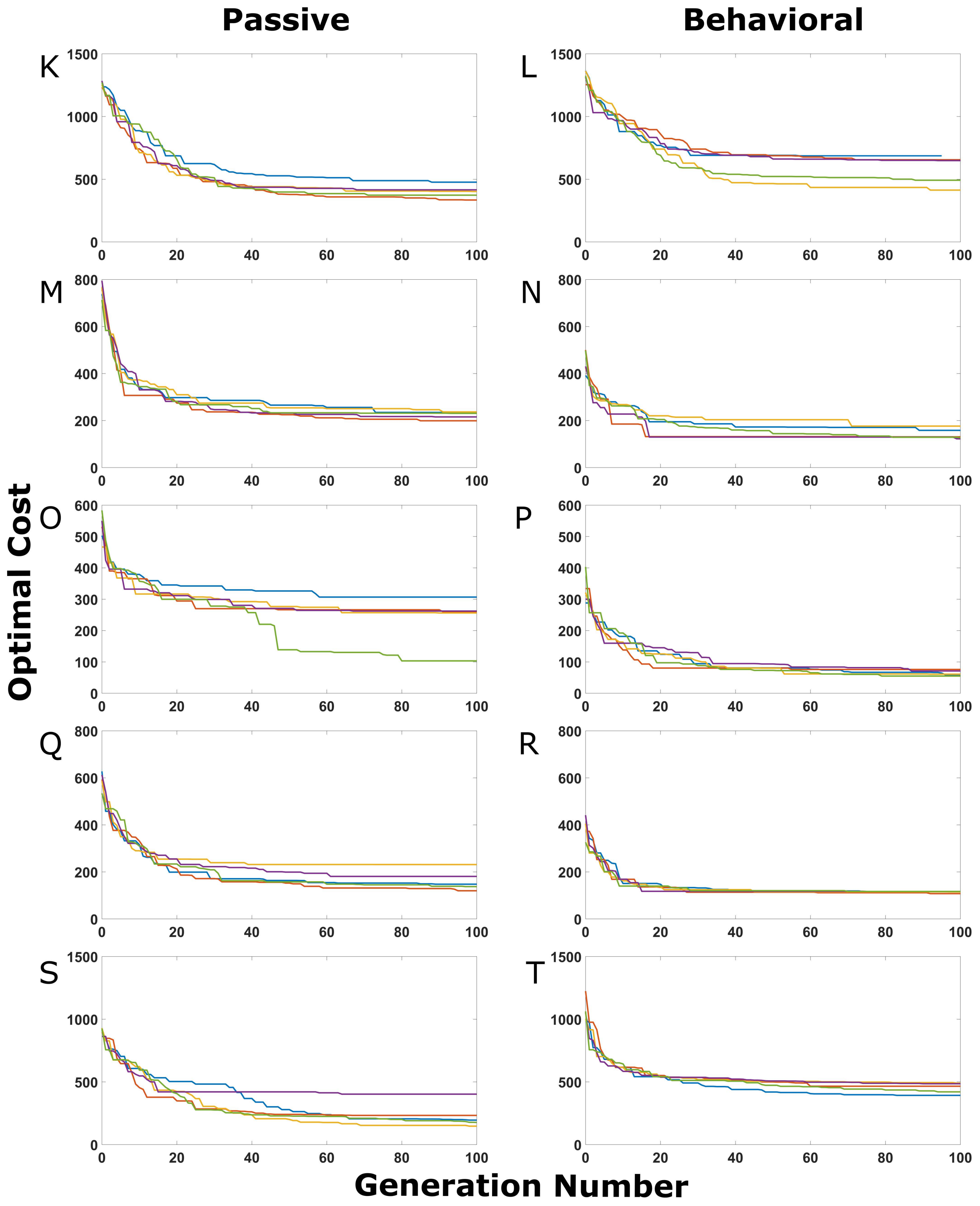

Passive
Behavioral
K
L
M
N
O
P
Q
R
S
T
Optimal Cost
Generation Number

Figure S1: Optimal value of the cost function for each generation of the genetic algorithm. Each row represents the output from the mathematical model for each of the ten single unit recordings. Panels on the left are optimised for the passive state, while panels on the right are optimised for the behavioral state. Each panel contains five lines, indicating the optimal value of the cost function for each repetition of the optimization for the same physiological data.
