# Supplementary material for: Computational Neural Modeling of Auditory Cortical Receptive Fields
Source: Front Comput Neurosci. 2019 May 24;13:28. doi: 10.3389/fncom.2019.00028 (PMC6543553; doi:10.3389/fncom.2019.00028)
Supplement: Supplementary file 2 [file Data_Sheet_2.pdf]

Cell 1

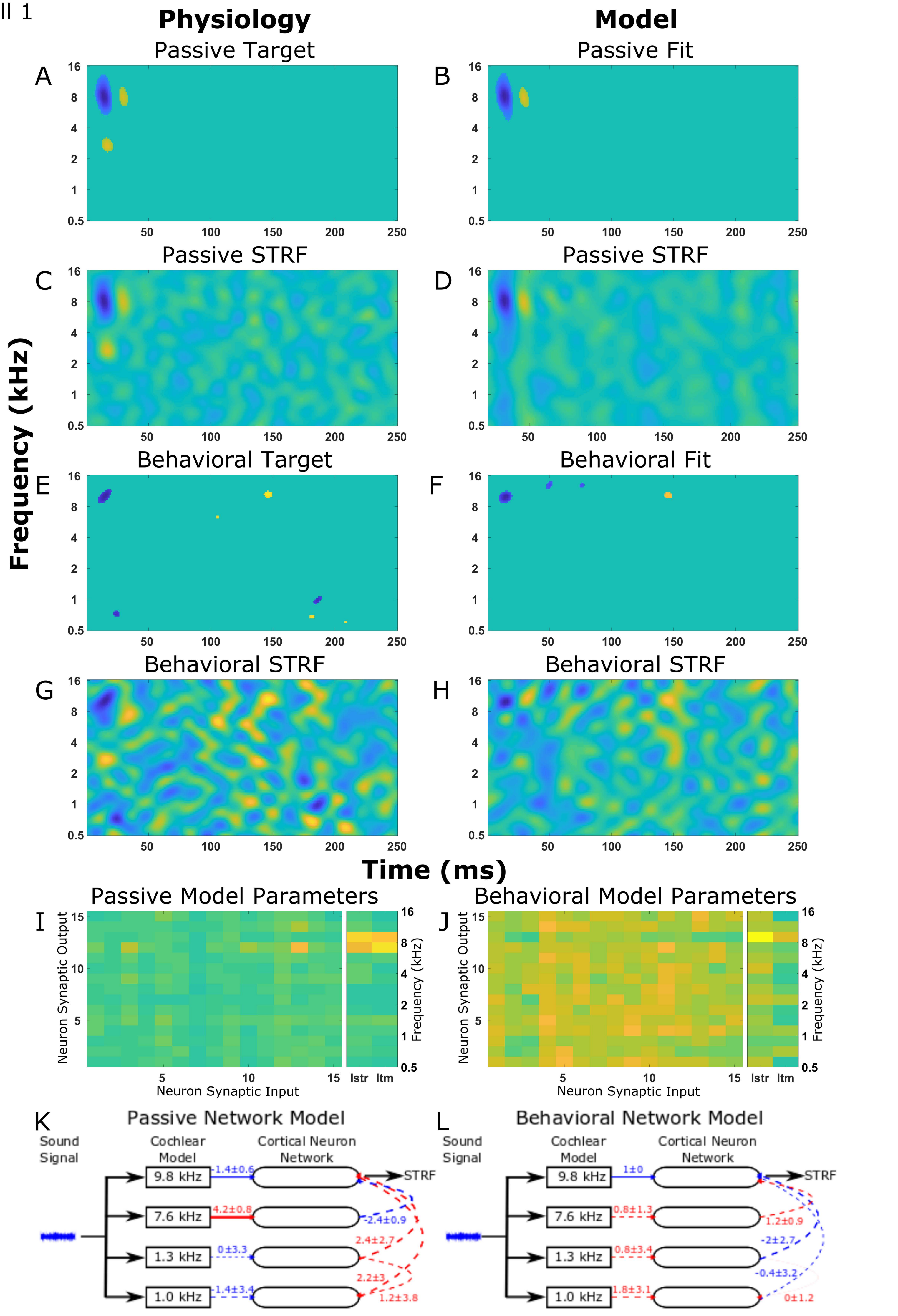
Physiology
Model
Passive Target
Passive Fit
Passive STRF
Passive STRF
Behavioral Target
Behavioral Fit
Behavioral STRF
Behavioral STRF
A
B
C
D
E
F
G
H
Frequency (kHz)
Time (ms)
Passive Model Parameters
Behavioral Model Parameters
I
J
Neuron Synaptic Output
Neuron Synaptic Input
Frequency (kHz)
Istr
Itm
K
L
Passive Network Model
Behavioral Network Model
Sound Signal
Cochlear Model
Cortical Neuron Network
STRF
9.8 kHz
7.6 kHz
1.3 kHz
1.0 kHz
1.4±0.6
4.2±0.8
0±3.3
1.4±3.4
-2.4±0.9
2.4±2.7
2.2±3
1.2±3.8
1±0
0.8±1.3
0.8±3.4
1.8±3.1
1.2±0.9
-2±2.7
-0.4±3.2
0±1.2

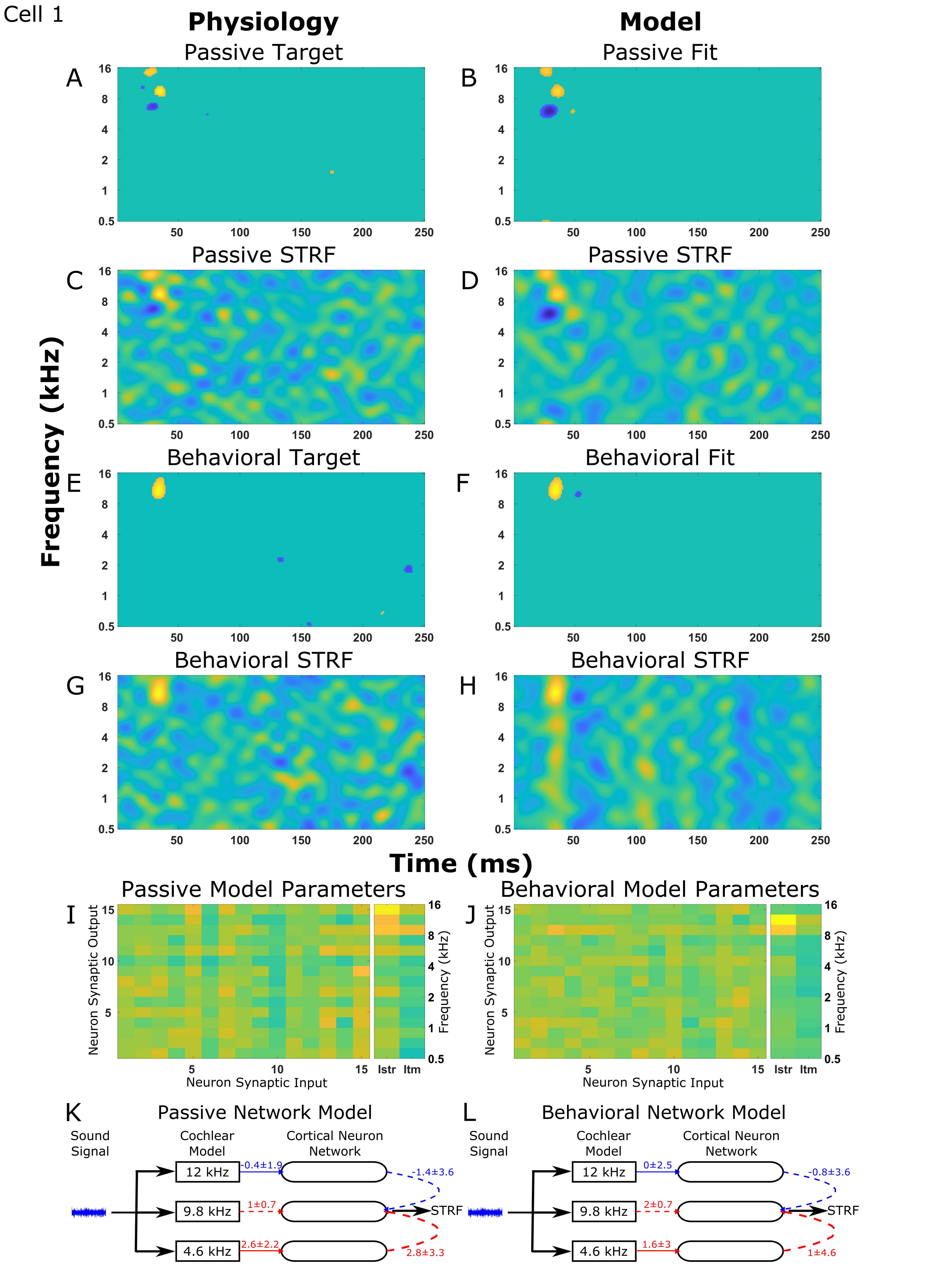

Cell 1
Physiology
Model
Passive Target
Passive Fit
Passive STRF
Behavioral Target
Behavioral Fit
Behavioral STRF
Frequency (kHz)
Time (ms)
Passive Model Parameters
Behavioral Model Parameters
Neuron Synaptic Output
Neuron Synaptic Input
Istr
Itm
Passive Network Model
Behavioral Network Model
Sound Signal
Cochlear Model
Cortical Neuron Network
12 kHz
9.8 kHz
4.6 kHz
STRF
-0.4±1.9
1±0.7
2.6±2.2
-1.4±3.6
2.8±3.3
0±2.5
2±0.7
1.6±3
-0.8±3.6
1±4.6

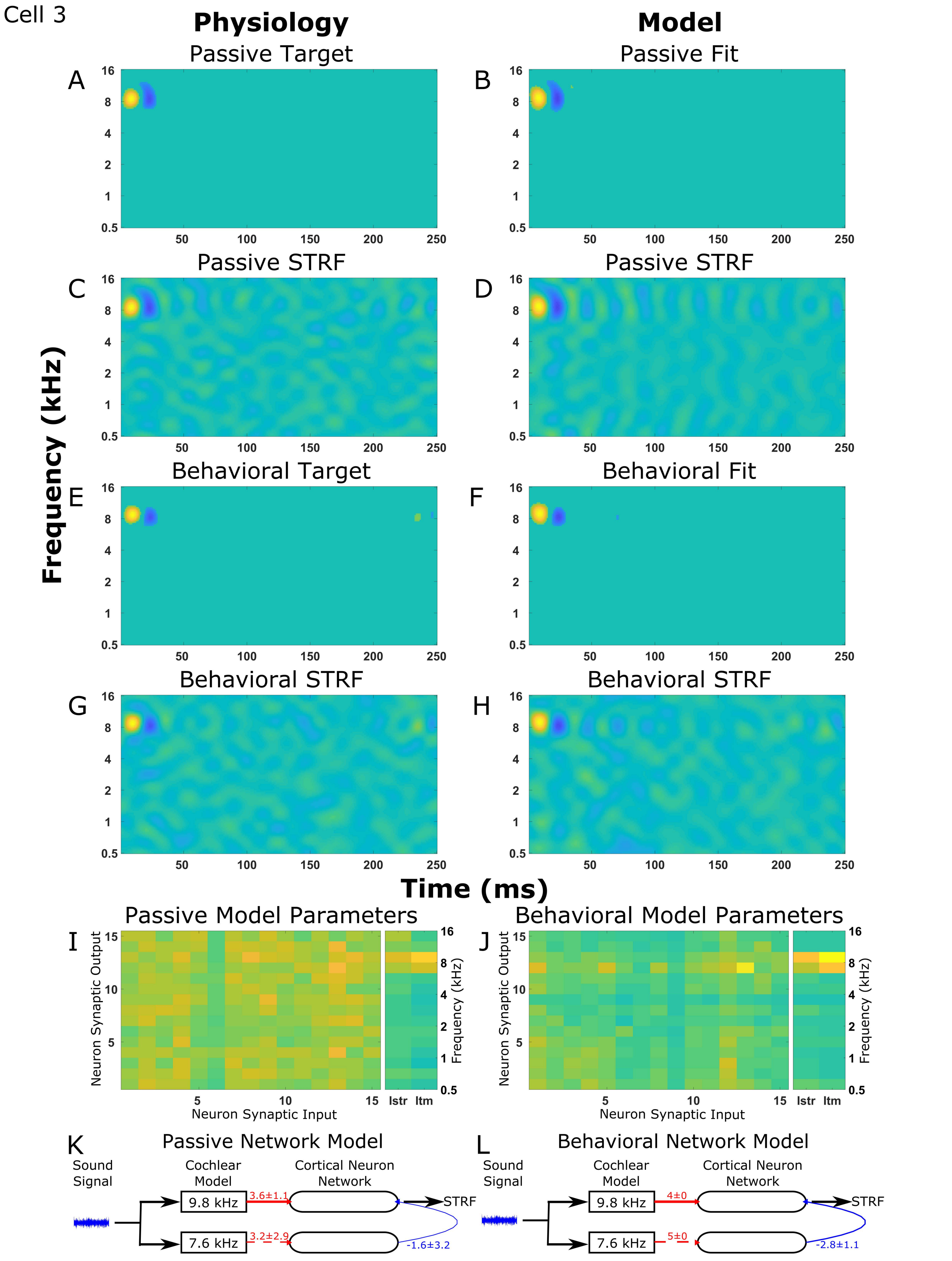

Cell 3
Physiology
Model
Passive Target
Passive Fit
Passive STRF
Passive STRF
Behavioral Target
Behavioral Fit
Behavioral STRF
Behavioral STRF
A
B
C
D
E
F
G
H
Frequency (kHz)
16
8
4
2
1
0.5
50
100
150
200
250
Time (ms)
Passive Model Parameters
Behavioral Model Parameters
I
J
Neuron Synaptic Output
Neuron Synaptic Input
Istr
Itm
Frequency (kHz)
K
Passive Network Model
L
Behavioral Network Model
Sound Signal
Cochlear Model
Cortical Neuron Network
9.8 kHz
7.6 kHz
3.6±1.1
3.2±2.9
-1.6±3.2
4±0
5±0
-2.8±1.1
STRF

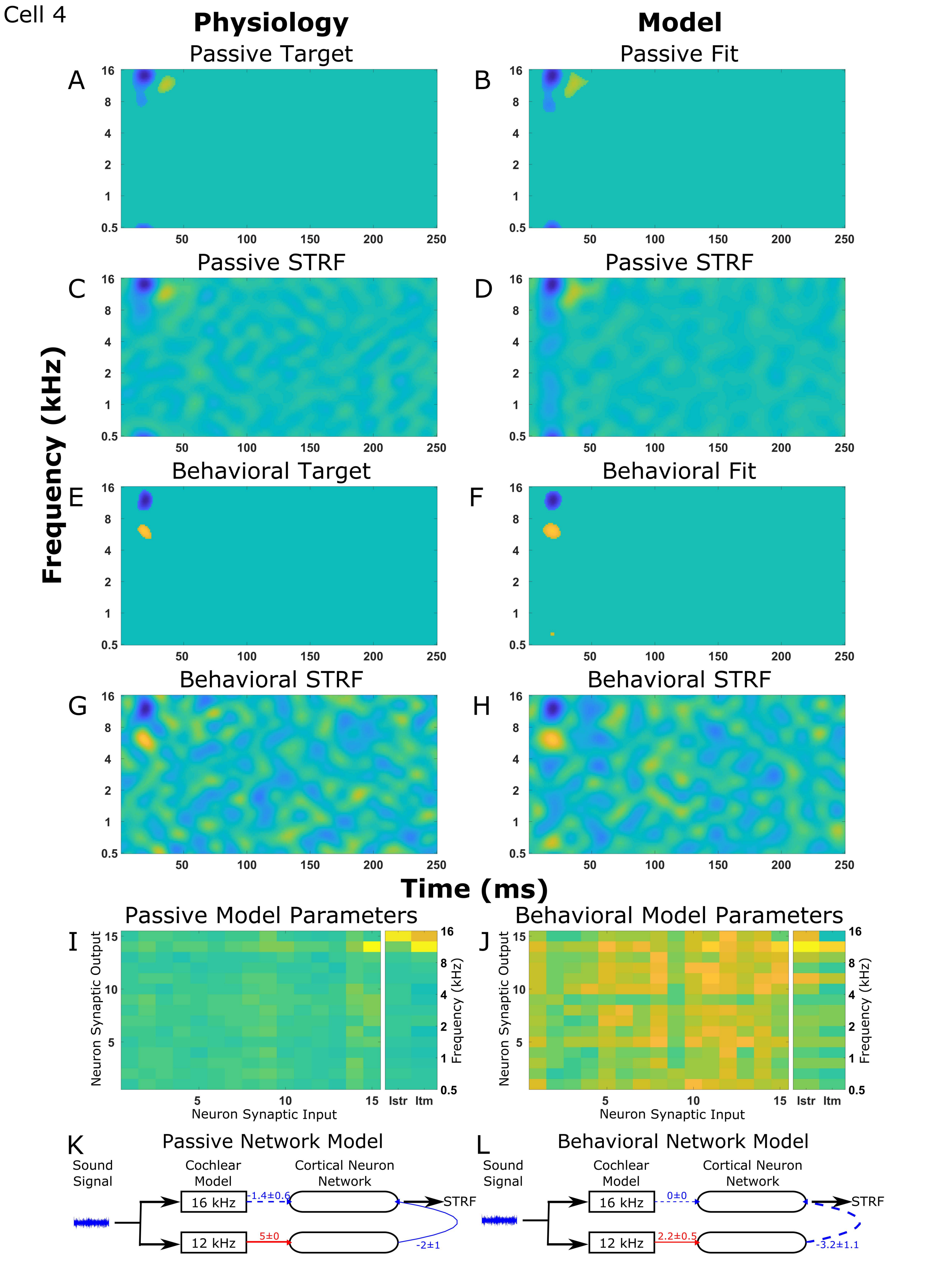

Cell 4
Physiology
Model
Passive Target
Passive Fit
A
B
Passive STRF
C
D
Behavioral Target
Behavioral Fit
E
F
Behavioral STRF
G
H
Frequency (kHz)
16
8
4
2
1
0.5
50
100
150
200
250
Time (ms)
Passive Model Parameters
Behavioral Model Parameters
I
J
Neuron Synaptic Output
Neuron Synaptic Input
Istr
Itm
Frequency (kHz)
K
Passive Network Model
L
Behavioral Network Model
Sound Signal
Cochlear Model
Cortical Neuron Network
16 kHz
12 kHz
STRF
-1.4±0.6
5±0
-2±1
0±0
2.2±0.5
-3.2±1.1

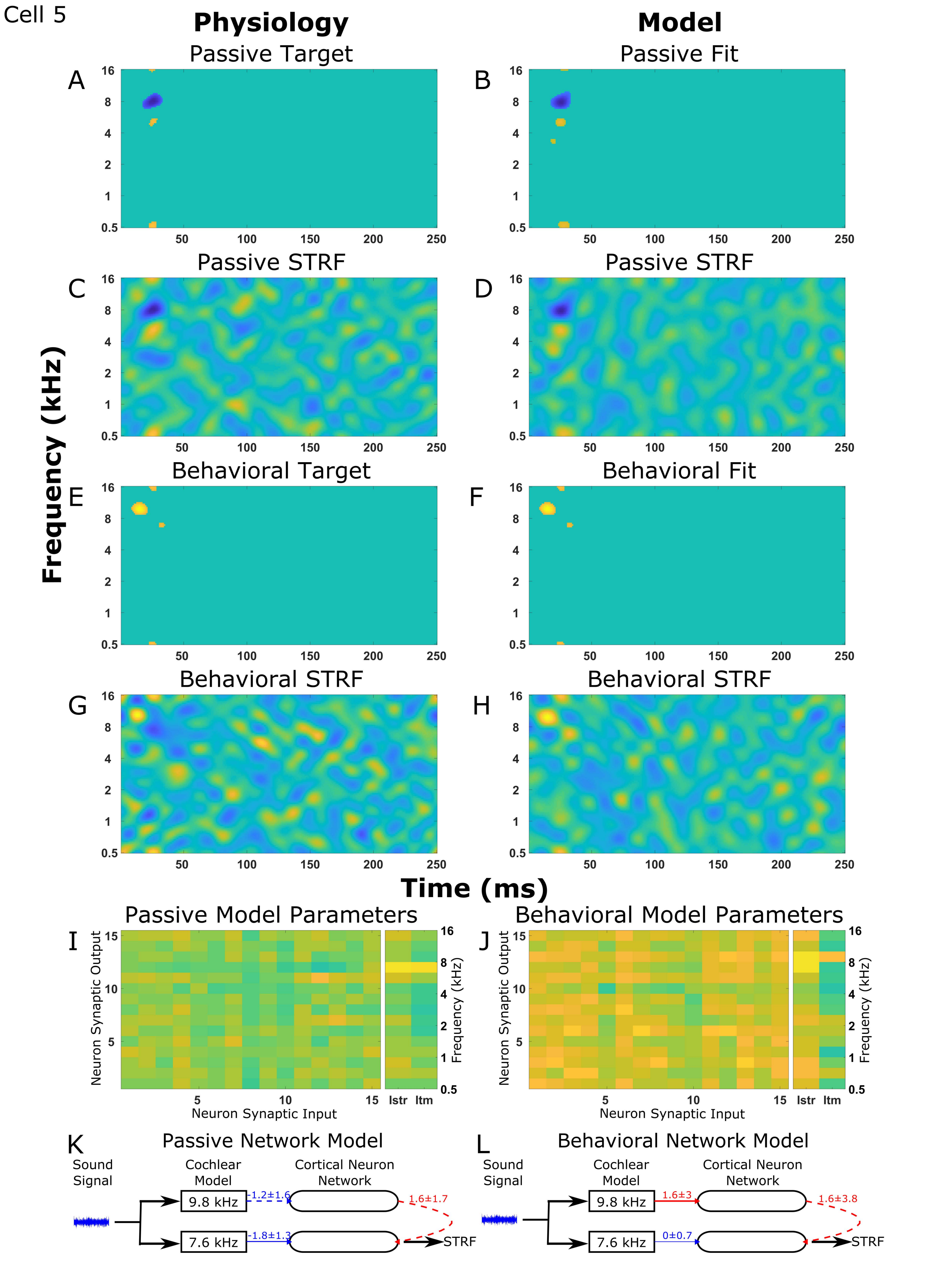
Cell 5
Physiology
Model
Passive Target
Passive Fit
Passive STRF
Passive STRF
Behavioral Target
Behavioral Fit
Behavioral STRF
Behavioral STRF
Frequency (kHz)
Time (ms)
Passive Model Parameters
Behavioral Model Parameters
Neuron Synaptic Output
Neuron Synaptic Input
Istr
Itm
Frequency (kHz)
Passive Network Model
Behavioral Network Model
Sound Signal
Cochlear Model
Cortical Neuron Network
9.8 kHz
7.6 kHz
-1.2±1.6
-1.8±1.3
1.6±1.7
1.6±3
0±0.7
1.6±3.8
STRF

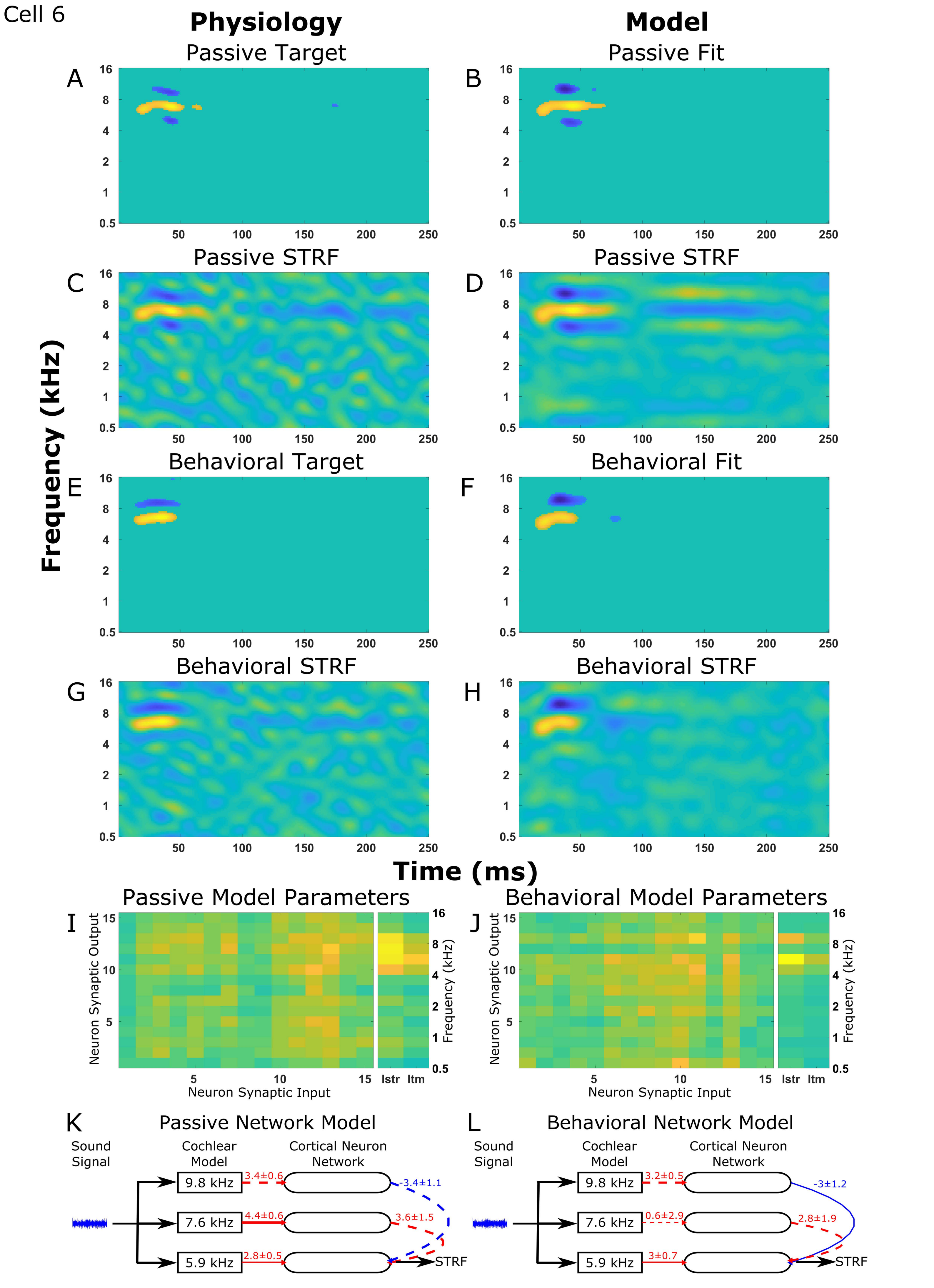
Cell 6
Physiology
Model
Passive Target
Passive Fit
Passive STRF
Passive STRF
Behavioral Target
Behavioral Fit
Behavioral STRF
Behavioral STRF
Frequency (kHz)
Time (ms)
Passive Model Parameters
Behavioral Model Parameters
Neuron Synaptic Output
Neuron Synaptic Input
Istr
Itm
Frequency (kHz)
Passive Network Model
Behavioral Network Model
Sound Signal
Cochlear Model
Cortical Neuron Network
9.8 kHz
7.6 kHz
5.9 kHz
3.4±0.6
4.4±0.6
2.8±0.5
-3.4±1.1
3.6±1.5
3.2±0.5
0.6±2.9
3±0.7
-3±1.2
2.8±1.9
STRF

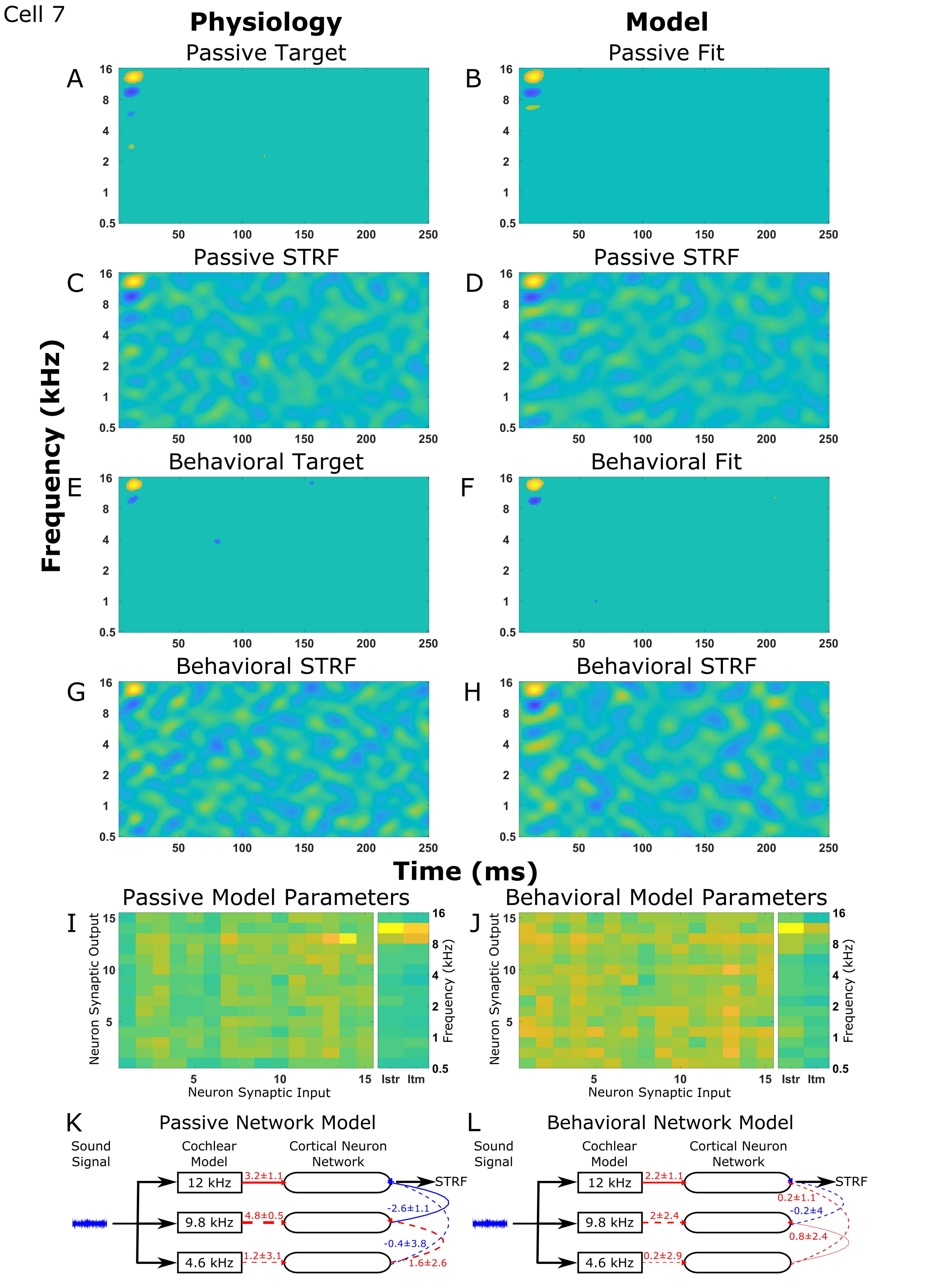
Cell 7
Physiology
Model
Passive Target
Passive Fit
A
B
C
D
E
F
G
H
Passive STRF
Passive STRF
Behavioral Target
Behavioral Fit
Behavioral STRF
Behavioral STRF
Frequency (kHz)
16
8
4
2
1
0.5
50
100
150
200
250
Time (ms)
I
J
Passive Model Parameters
Behavioral Model Parameters
Neuron Synaptic Output
Neuron Synaptic Input
Frequency (kHz)
Istr
Itm
K
L
Passive Network Model
Behavioral Network Model
Sound Signal
Cochlear Model
Cortical Neuron Network
12 kHz
9.8 kHz
4.6 kHz
STRF
3.2±1.1
4.8±0.5
1.2±3.1
-2.6±1.1
-0.4±3.8
1.6±2.6
2.2±1.1
2±2.4
0.2±2.9
0.2±1.1
-0.2±4
0.8±2.4

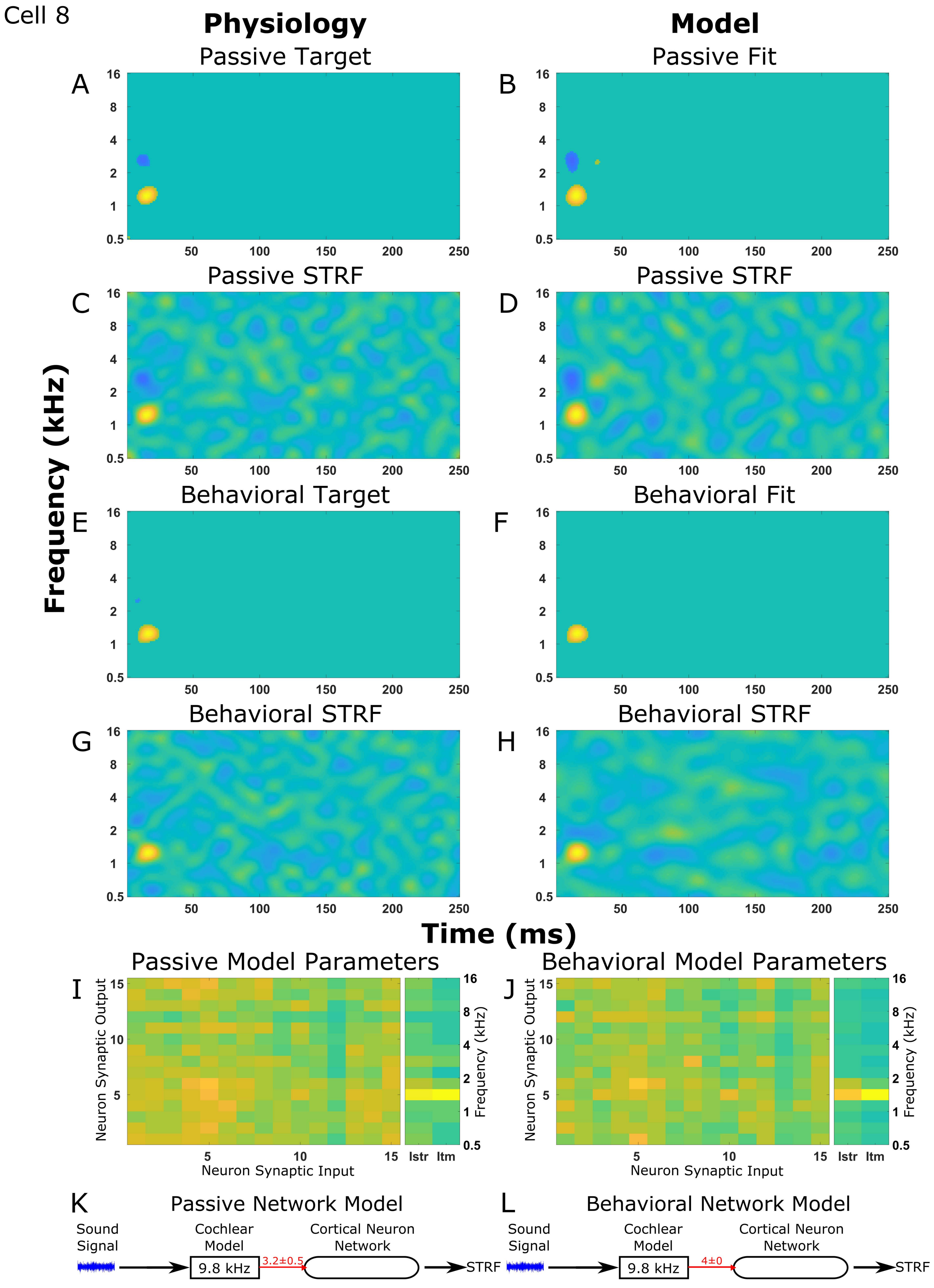
Cell 8
Physiology
Model
Passive Target
Passive Fit
Passive STRF
Passive STRF
Behavioral Target
Behavioral Fit
Behavioral STRF
Behavioral STRF
Frequency (kHz)
Time (ms)
Passive Model Parameters
Behavioral Model Parameters
Neuron Synaptic Output
Neuron Synaptic Input
Istr
Itm
Frequency (kHz)
Passive Network Model
Behavioral Network Model
Sound Signal
Cochlear Model
Cortical Neuron Network
9.8 kHz
3.2±0.5
4±0
STRF

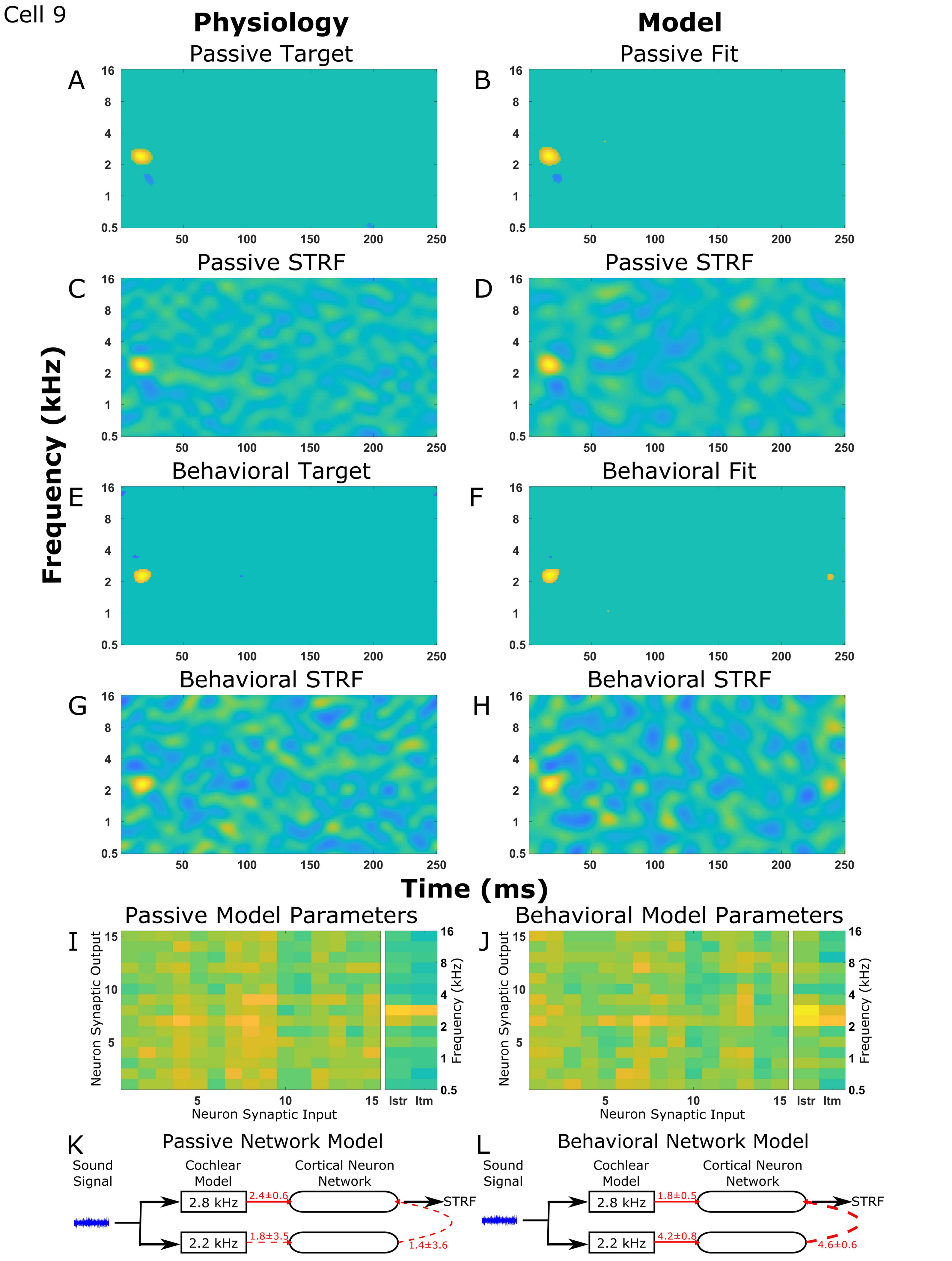

Cell 9
Physiology
Model
Passive Target
Passive Fit
Passive STRF
Passive STRF
Behavioral Target
Behavioral Fit
Behavioral STRF
Behavioral STRF
A
B
C
D
E
F
G
H
Frequency (kHz)
16
8
4
2
1
0.5
50
100
150
200
250
Time (ms)
Passive Model Parameters
Behavioral Model Parameters
I
J
Neuron Synaptic Output
Neuron Synaptic Input
Istr
Itm
Frequency (kHz)
K
Passive Network Model
L
Behavioral Network Model
Sound Signal
Cochlear Model
Cortical Neuron Network
2.8 kHz
2.2 kHz
STRF
2.4±0.6
1.8±3.5
1.4±3.6
1.8±0.5
4.2±0.8
4.6±0.6

Cell 10

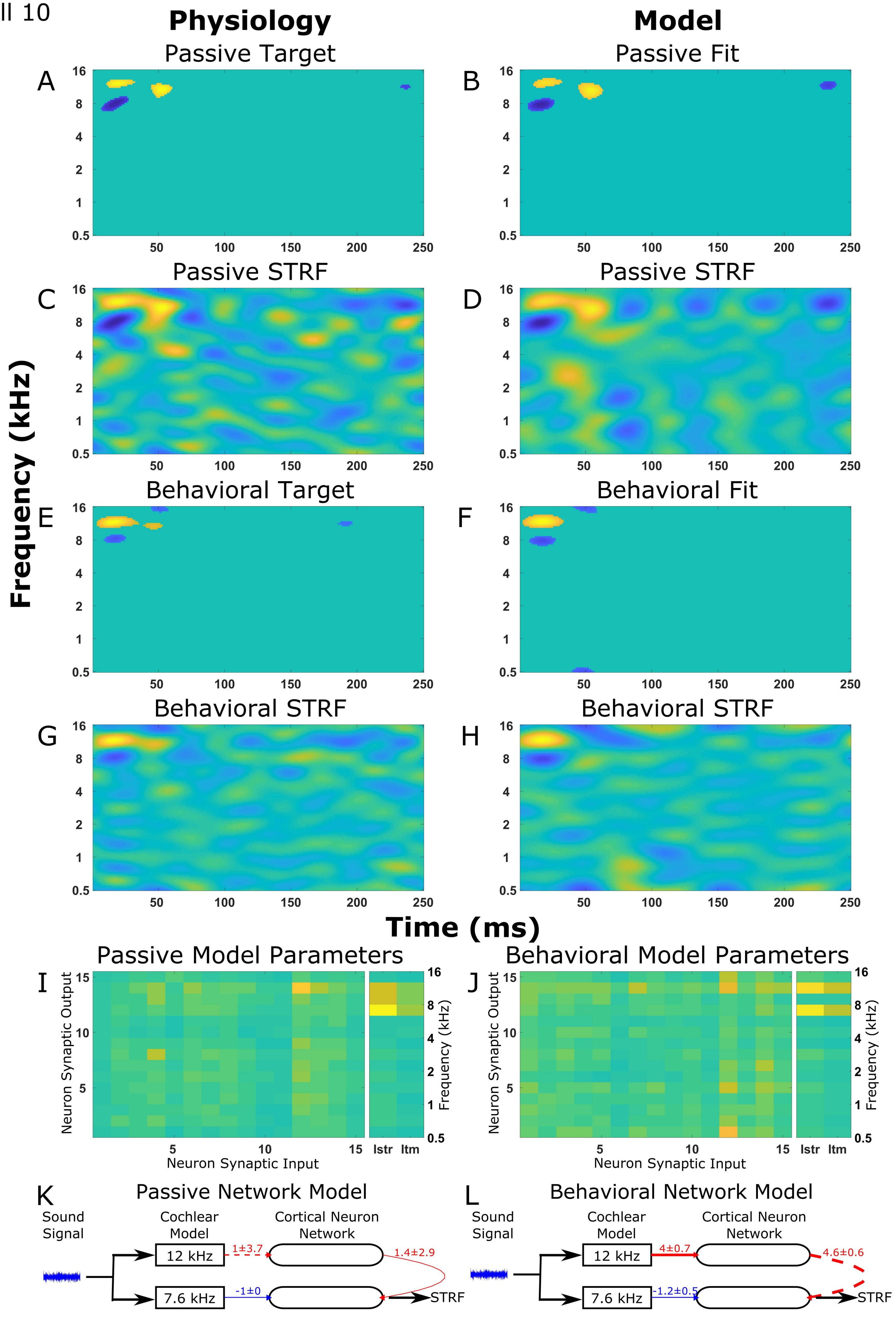
Physiology
Model
Passive Target
Passive Fit
A
B
Passive STRF
Passive STRF
C
D
Behavioral Target
Behavioral Fit
E
F
Behavioral STRF
Behavioral STRF
G
H
Frequency (kHz)
16
8
4
2
1
0.5
50
100
150
200
250
Time (ms)
Passive Model Parameters
Behavioral Model Parameters
I
J
Neuron Synaptic Output
Neuron Synaptic Input
Frequency (kHz)
Istr
Itm
K
Passive Network Model
L
Behavioral Network Model
Sound Signal
Cochlear Model
Cortical Neuron Network
12 kHz
7.6 kHz
1±3.7
-1±0
1.4±2.9
STRF
4±0.7
-1.2±0.5
4.6±0.6

Figure S2: STRF and changes in the network structure for all ten single unit recordings. Each page represents the physiological recordings and output from the mathematical model for each single unit recording. Physiological recordings were used to optimize the synaptic drive in a neural network model and sensitivity analyses of network structures were performed to highlight important parameters of ten single unit recordings (**A-D)**. Within each panel, the first two columns display the regions of the STRF that were significantly different (greater than 3 standard deviations) from the mean value of the STRF. The first column is for the passive state; the second column is the behavioral state. The first row of STRFs is electrophysiological recordings, while the second row is the outputs from the model. The third column displays the important network parameters for the passive and behavioral states. In the network schematic diagrams, solid lines indicate that parameters have a high sensitivity, whereas dashed lines indicate the parameter was not sensitive but is provided for comparison between the two network structures or to follow the pathway from sound signal to the neuron from which the STRF is calculated. Red lines indicate excitatory synaptic connections, blue lines indicate inhibitory synaptic connections, and the thicknesses of the lines indicate the strengths of the synaptic connections. The numerical values presented for each line indicate the mean ± standard deviation for the five repetitions of the optimization.
