# Supplementary material for: Computational Neural Modeling of Auditory Cortical Receptive Fields
Source: Front Comput Neurosci. 2019 May 24;13:28. doi: 10.3389/fncom.2019.00028 (PMC6543553; doi:10.3389/fncom.2019.00028)
Supplement: Supplementary file 3 [file Data_Sheet_3.PDF]

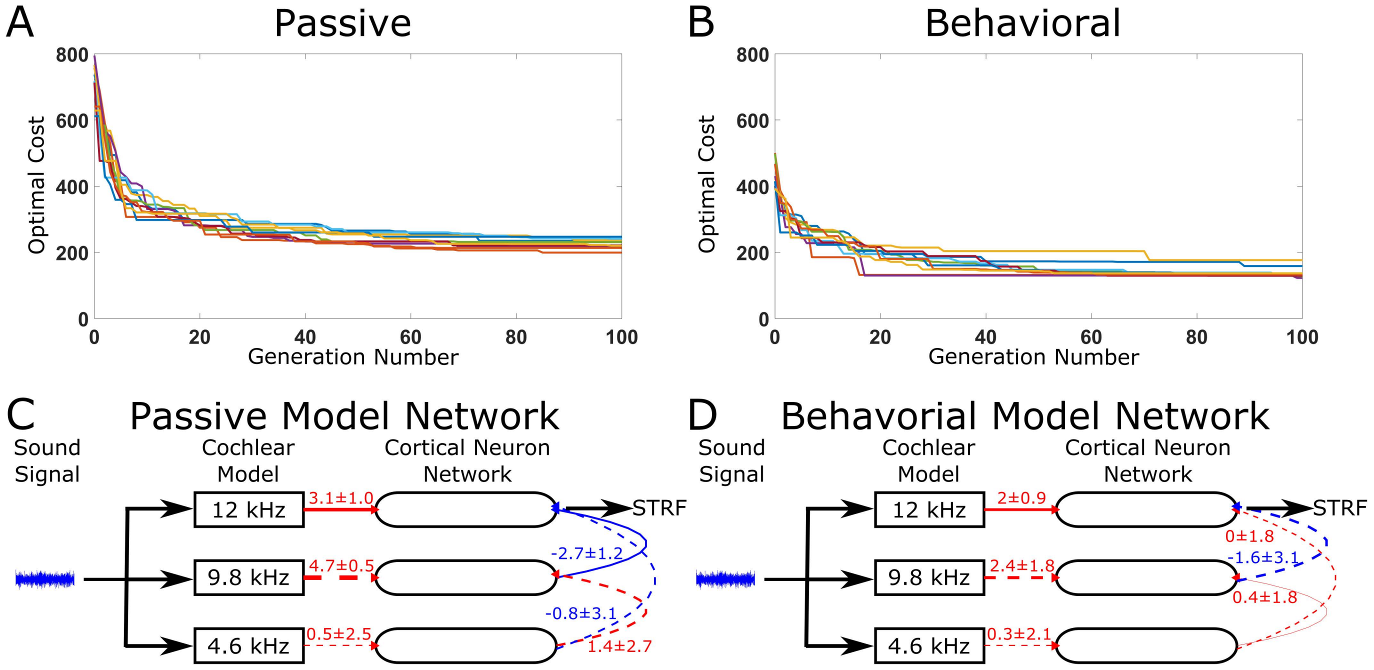

Figure S3: Changes in the network structure for 10 repetitions. The mathematical model can reproduce physiological recordings from the passive and behavioral states (Figure 4) and changes in the network structure were identified over five repetitions (Figure 5). For this figure, the number of repetitions was increased to ten. Panels **A** and **B** show the optimal value of the cost function for each generation of the genetic algorithm. Panels **C** and **D** show the network structure and can be directly compared to panels C and D in Figure 5.
